# QSAR-ML- and Metadynamics-Guided Design of Symmetrical Bis-Indanones to Overcome Mutational Anchor Loss in Acetylcholinesterase

**DOI:** 10.3390/ph19081169

**Published:** 2026-07-26

**Authors:** Ghazala Muteeb, Shrikant S. Nilewar, Mohammad Aatif, Tushar Janardan Pawar

**Affiliations:** 1 Department of Nursing, College of Applied Medical Sciences, King Faisal University, Al-Ahsa 31982, Saudi Arabia; graza@kfu.edu.sa; 2 Department of Pharmaceutical Chemistry, Maliba Pharmacy College, Uka Tarsadia University, Bardoli 394350, Gujarat, India; shrinilewar@gmail.com; 3 Department of Public Health, College of Applied Medical Sciences, King Faisal University, Al-Ahsa 31982, Saudi Arabia; maahmad@kfu.edu.sa; 4 Centro de Investigación, Universidad Anáhuac Querétaro, Circuito Universidades I, Fracción 2 S/N, Zibatá, El Marqués 76246, Querétaro, Mexico

**Keywords:** acetylcholinesterase, symmetrical probes, QSAR-ML validation, metadynamics, allosteric rewiring, solvation rescue, thermodynamic locking, neurotherapeutics

## Abstract

**Background/Objectives:** Symmetrical dual-site acetylcholinesterase (AChE) inhibitors offer a compelling strategy to mitigate mutational drug resistance, yet static modeling fails to capture induced-fit dynamics under mutational stress. **Methods:** Here, a 100,000-compound virtual library was filtered using a machine learning-based QSAR classification pipeline. A strict, empirically calibrated Jaccard applicability domain filter (AD = 0.823) eliminated topological anomalies, yielding a robust cross-validation accuracy (ROC-AUC: 0.80 ± 0.05; independent test MCC: 0.61). Multi-parameter ADMET and shape screening prioritized unique chemotypes to probe the 20 Å enzyme gorge. All-atom explicit-solvent molecular dynamics simulations were coupled with 150 ns enhanced-sampling Metadynamics along two orthogonal collective variables (gorge depth and ligand orientation) to map out the free energy surfaces under mutational stress. **Results:** Symmetrical probes suffered catastrophic unbinding upon anchor loss. Conversely, the symmetrical core of Lead Compound **1631** demonstrated extraordinary structural resilience. In silico site-directed mutagenesis (W86A and W286A) triggered a thermodynamic locking effect; the W86A mutant forced the complex into a deeper energetic well (ΔG_min_ = 9.23 ± 1.98 kJ/mol) than the wild-type state (5.26 ± 1.69 kJ/mol). MM/GBSA decomposition confirmed an active electrostatic-solvation compensation mechanism along a “solvation see-saw” diagonal (ΔΔG_total_ = +1.59 kcal/mol). Finally, Dynamic Cross-Correlation Matrix analysis quantified a mechanical inversion of the CAS-PAS axis into an anti-correlated clamping mode (−0.04) that locked the ligand bridge in place. **Conclusions:** These results demonstrate that symmetrical dual-site targeting, combined with dynamic thermodynamic locking, provides a resilient framework to overcome mutational resistance in AChE inhibitors.

## 1. Introduction

The modern shift toward an increasingly aging global population has brought about a critical neuropharmacological challenge, characterized by the widespread rise of Alzheimer’s disease (AD) as the primary cause of dementia across the globe. Currently representing between 60% and 70% of the 55 million individuals diagnosed with dementia worldwide, the total case burden of AD is expected to reach nearly 139 million by the middle of the century [1,2,3,4]. This rapid growth carries severe socioeconomic risks; the international financial burden generated by dementia care, estimated at USD 1.3 trillion in 2020, is on track to double to USD 2.8 trillion by 2030, a trajectory that threatens to overwhelm healthcare infrastructures in both developed and emerging economies [3,4]. This trend is particularly clear in high-growth demographic areas such as India, where the localized patient population is expected to surpass 14 million by 2040, reinforcing the critical demand for scalable, highly effective medical options. Yet, despite multi-decade research efforts, clinical management continues to rely almost entirely on transient, palliative measures that do not address the progressive neurodegenerative process, leaving a distinct gap in disease-modifying therapies [5,6].

The clinical disillusionment following the controversial approval and subsequent efficacy concerns of amyloid-targeted monoclonal antibodies has highlighted the inherent limitations of mono-target paradigms [7,8]. These high-cost biological therapies have been plagued by significant safety issues, specifically Amyloid-Related Imaging Abnormalities (ARIA) [9,10]. Furthermore, pharmacovigilance data establishing concerning associations between common medications and increased dementia events underscore the necessity for novel therapeutics that possess superior safety profiles and high-occupancy target engagement [11,12]. Consequently, scientific interest has been reinvigorated in the cholinergic hypothesis, repositioning the enzyme acetylcholinesterase (AChE) as a multifunctional structural target [13,14]. While AChE typically facilitates the rapid hydrolysis of the neurotransmitter acetylcholine (ACh) within the synaptic cleft, the AD brain suffers from a profound ACh deficit compounded by sustained enzymatic activity [15,16].

The structural anatomy of the human AChE enzyme provides a sophisticated, albeit challenging, blueprint for drug design. The enzyme contains a narrow, 20 Å deep active-site gorge with two distinct binding domains: the Catalytic Active Site (CAS) at the base and the Peripheral Anionic Site (PAS) at the entrance. Traditional inhibitors primarily target the CAS to provide transient symptomatic relief [17,18,19]. However, the PAS serves as more than a gatekeeper; it acts as a pro-aggregating modulator of β-amyloid (Aβ) peptide aggregation. The interaction between the PAS and Aβ peptides accelerates the formation of neurotoxic fibrils, creating a pathological bridge between cholinergic failure and the amyloidogenic cascade [20,21]. This dual functionality makes AChE a pivotal target for dual-site inhibitors, molecules designed to simultaneously anchor at the CAS to preserve ACh and blockade the PAS to halt amyloid aggregation [22,23].

Despite the conceptual elegance of dual-site inhibition within the 20 Å hAChE gorge, a critical pharmacological vulnerability persists. Most historical dual-site scaffolds are structurally asymmetric, relying on distinct, single-pole interactions at the catalytic active site (CAS) and the peripheral anionic site (PAS) [24,25]. Consequently, single-point target-site mutations within the channel can induce severe therapeutic stagnation or catastrophic ligand unbinding due to the loss of a single critical mutational anchor [26]. Literature reveals a striking lack of structural engineering strategies focused on deploying molecular symmetry to achieve binding redundancy within the gorge. Symmetrical architectures present a compelling solution to evolutionary target modifications; by featuring duplicated pharmacophoric poles, a symmetrical ligand can theoretically distribute its thermodynamic load and maintain target occupancy even when a primary amino acid anchor is lost [27].

In the present work, we address these multifaceted challenges through a rigorous computational optimization pipeline grounded in machine learning validation and enhanced physics-based dynamic simulations. Moving away from unvalidated generative abstractions, we deploy a robust QSAR-ML classification framework to evaluate a high-dimensional chemical space, enforcing a strict, empirically calibrated Jaccard Applicability Domain (AD) filter to eliminate topological anomalies and ensure structural viability. Symmetrical bis-indanone derivatives designed under these topological constraints were subjected to rigorous multi-parameter ADMET screening to ensure optimal blood–brain barrier (BBB) penetration and lead-like druglikeness. Crucially, we transition from static docking to sequential 100 ns explicit-solvent molecular dynamics (MD) and 150 ns Metadynamics enhanced-sampling simulations to stress-test these architectures under simulated site-directed mutations (W86A and W286A). Through Molecular Mechanics/Generalized Born Surface Area (MM/GBSA) free energy calculations and Dynamic Cross-Correlation Matrix (DCCM) analysis, we map the structural stability, thermodynamic landscapes, and mechanical coupling shifts in these complexes. This study provides a validated biophysical blueprint for the discovery of mutation-resistant neurotherapeutics, demonstrating how symmetrical scaffolds can actively leverage enzyme plasticity to achieve solvation rescue and allosteric rewiring across evolutionary variants.

## 2. Results and Discussion

### 2.1. Chemoinformatic Library Screening, QSAR-ML Validation, and Applicability Domain Triage

**Figure 1:**
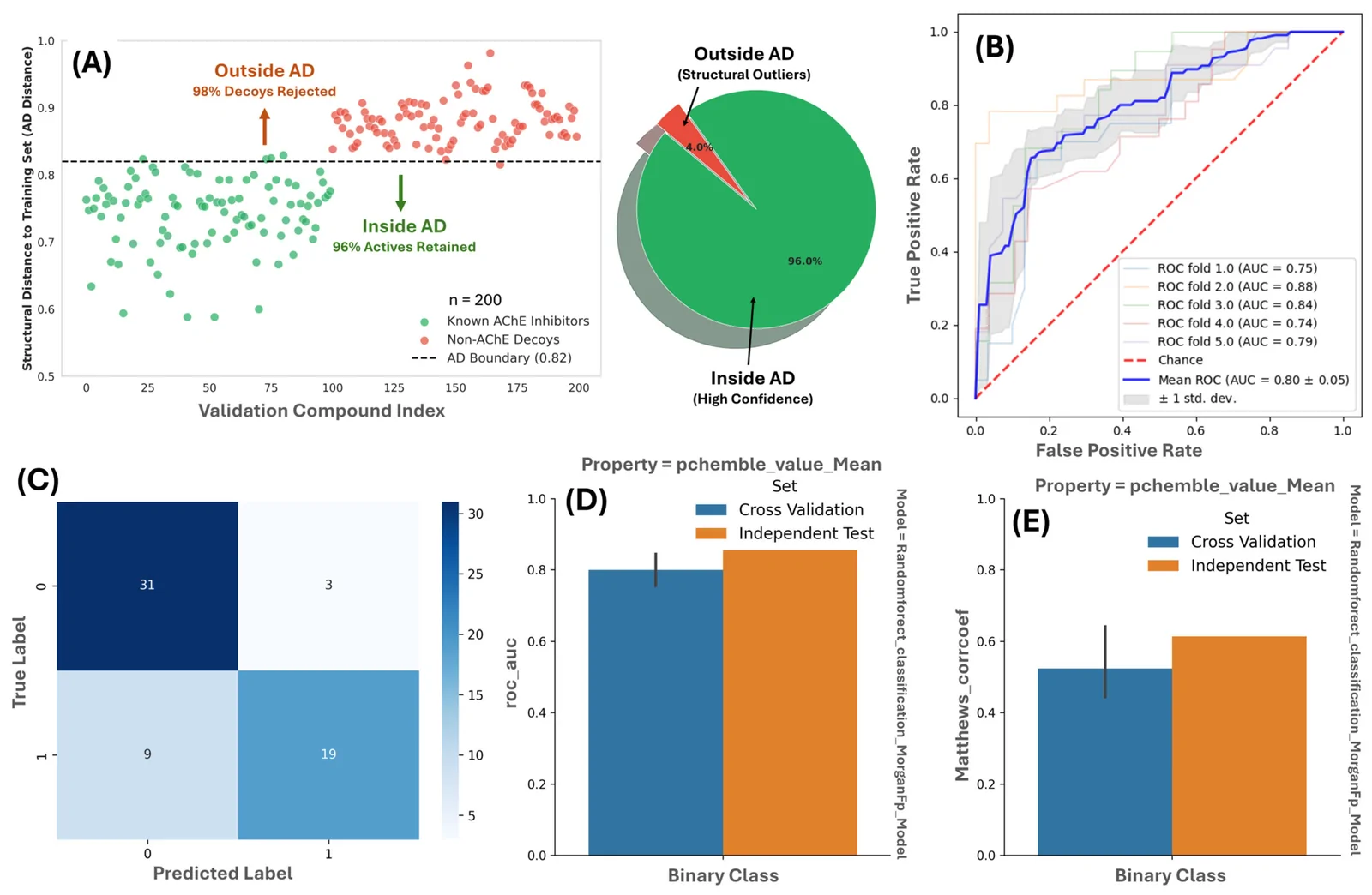
Machine learning validation and applicability domain calibration metrics for the hAChE classification pipeline. (**A**) Jaccard distance distribution matrix mapping active inhibitors and commercial decoys relative to the 0.823 applicability domain boundary. (**B**) Five-fold cross-validation receiver operating characteristic (ROC) trajectories yielding a mean AUC of 0.80 ± 0.05. (**C**) Two-dimensional confusion matrix quantifying true and predicted binary classifications for the independent test set. (**D**) Comparative histogram tracking ROC-AUC values across internal cross-validation and independent validation distributions. (**E**) Matthews Correlation Coefficient (MCC) values calculated across the primary validation layers.

Traditional computational drug discovery workflows and virtual screening campaigns frequently confine lead exploration to restricted, well-characterized chemical domains, potentially missing structurally distinct or mutationally resilient architectures. To mitigate this limitation, this study deployed an integrated machine learning-driven screening pipeline coupled with multi-parameter property filters to safely navigate unexplored areas of the hAChE chemical space. Initial predictive scoring functions were established by training a Random Forest classification architecture. Evaluated via a rigorous stratified five-fold cross-validation protocol, the optimized QSAR baseline model demonstrated high statistical robustness, achieving a mean receiver operating characteristic area under the curve (ROC-AUC) of 0.80 ± 0.05 (Figure 1B).

Despite this predictive accuracy, high-dimensional screening models can be sensitive to topological outliers, producing structurally anomalous or chemically unviable configurations that artificially skew classification thresholds. To structurally safeguard the discovery pipeline, the predictive boundaries of the machine learning model were regulated by calibrating a strict geometric Applicability Domain (AD) using an external validation cohort of 200 characteristically balanced compounds. Distance distribution analysis mapping the multi-dimensional fingerprint space revealed a clear structural and biophysical separation between verified hAChE active inhibitors and diverse commercial decoys (Figure 1A). An optimal hard Jaccard dissimilarity threshold of 0.823 was mathematically established as the absolute boundary limit for model reliability (Figure 1A). At this specific threshold, the calibrated AD filter exhibited high discriminatory power, successfully identifying and rejecting 98% of the structural decoys while retaining 96% of the verified active control inhibitors, with only a minor 4% allocation of structural outliers falling outside the high-confidence domain (Figure 1A). The exclusion of 4% of known active compounds highlights the conservative nature of this boundary, sacrificing highly unusual or extreme structural outliers to secure the overall integrity of the predictive chemical space.

By deploying this calibrated threshold as an initial filter, a raw pool of 100,000 candidate configurations was constrained to the reliable structural space of the training data. This boundary enforcement guaranteed that all downstream topographical, kinetic, and thermodynamic evaluations were conducted exclusively on realistic and chemically viable small-molecule architectures.

To thoroughly validate the independent predictive performance of the scoring function within this regulated domain, the model was tested against an independent test set. The resulting classification accuracy was quantified using an explicit confusion matrix (Figure 1C), which confirmed highly accurate sample distribution with 31 true negatives and 19 true positives, balanced against a low rate of misclassifications consisting of 3 false positives and 9 false negatives. This balanced performance was supported by tracking the statistical metrics across both internal cross-validation and independent test distributions (Figure 1D). The absolute classification accuracy remained consistent, showing an independent test ROC-AUC value of 0.84, which successfully exceeded the cross-validation baseline (Figure 1D).

To confirm that the classifier maintained a highly reliable prediction rate free from fractional optimization bias, the Matthews Correlation Coefficient (MCC) was calculated across the evaluation layers (Figure 1E). The model achieved a stable cross-validation MCC baseline alongside a definitive independent test MCC value of 0.61 (Figure 1E). Analysis of the misclassification distribution (3 false positives and 9 false negatives) indicates a highly conservative predictive bias, which intentionally prioritizes the exclusion of structural outliers over absolute sensitivity. This structural validation proved that the machine learning setup possessed the statistical reliability required to guide downstream candidate prioritization.

Following the initial scoring and applicability domain enforcement, the surviving candidates were filtered through sequential multi-parameter ADMET and topological screens to ensure CNS compatibility. The structural geometry of the hAChE enzyme gorge presents a distinct biophysical barrier, requiring small molecules to maintain an optimal balance between spatial orientation and conformational flexibility. To address this structural constraint, a strict molecular shape index threshold (I_shape_ > 0.60) was enforced, filtering for elongated, linear topologies designed to effectively span the 20 Å distance between the peripheral entrance and the catalytic base without causing steric clashes. Concurrently, rotatable bond frequencies were capped between 6 and 8, providing the conformational flexibility necessary for induced-fit adaptations while minimizing the entropic penalties associated with macro-structural binding.

Compounds containing reactive toxophores or structural motifs associated with pan-assay interference (PAINS) were excluded to eliminate non-specific covalent aggregation and guarantee a completely reversible mechanism of action. To ensure that the resulting compounds were chemically tractable rather than theoretical artifacts, synthetic accessibility (SA) scores were systematically calculated, confirming that the prioritized configurations occupied an accessible score distribution ranging between 3.43 and 4.49.

This multi-step property triage reduced the virtual screening output to 536 highly qualified molecules. To remove lingering analog redundancy and expand the topological diversity of the final lead space, a leader-based Butina clustering algorithm was applied using a pairwise Tanimoto similarity threshold of 0.85. The clustering workflow eliminated 19 redundant structural analogs, yielding a final, highly optimized repository of 517 distinct candidate chemotypes. Based on the molecular docking results of the refined 517 compounds, four structurally distinct probes belonging to the alkylene-linked bis-indanone and amino-quinoline families were explicitly selected to map the functional extremes of the 20 Å gorge. These included Ligand **3191**, designated as the Surface Lid for its PAS-dependent binding; Ligand **1821**, the Deep Penetrator due to its strict CAS reliance; and Ligand **41**, the Non-canonical Disruptor chosen for its adaptive potential. Finally, the symmetrical dimer Lead Compound **1631** was prioritized as the Symmetrical Bridge. Because Lead Compound **1631** perfectly balances PAS and CAS engagement, it was established as the ultimate subject to undergo subsequent rigorous thermodynamic analyses to test how allosteric tension redistributes when a primary anchor is lost.

### 2.2. Structural Basis of Spatial Binding: Docking and Non-Covalent Interaction Profiling

**Figure 2:**
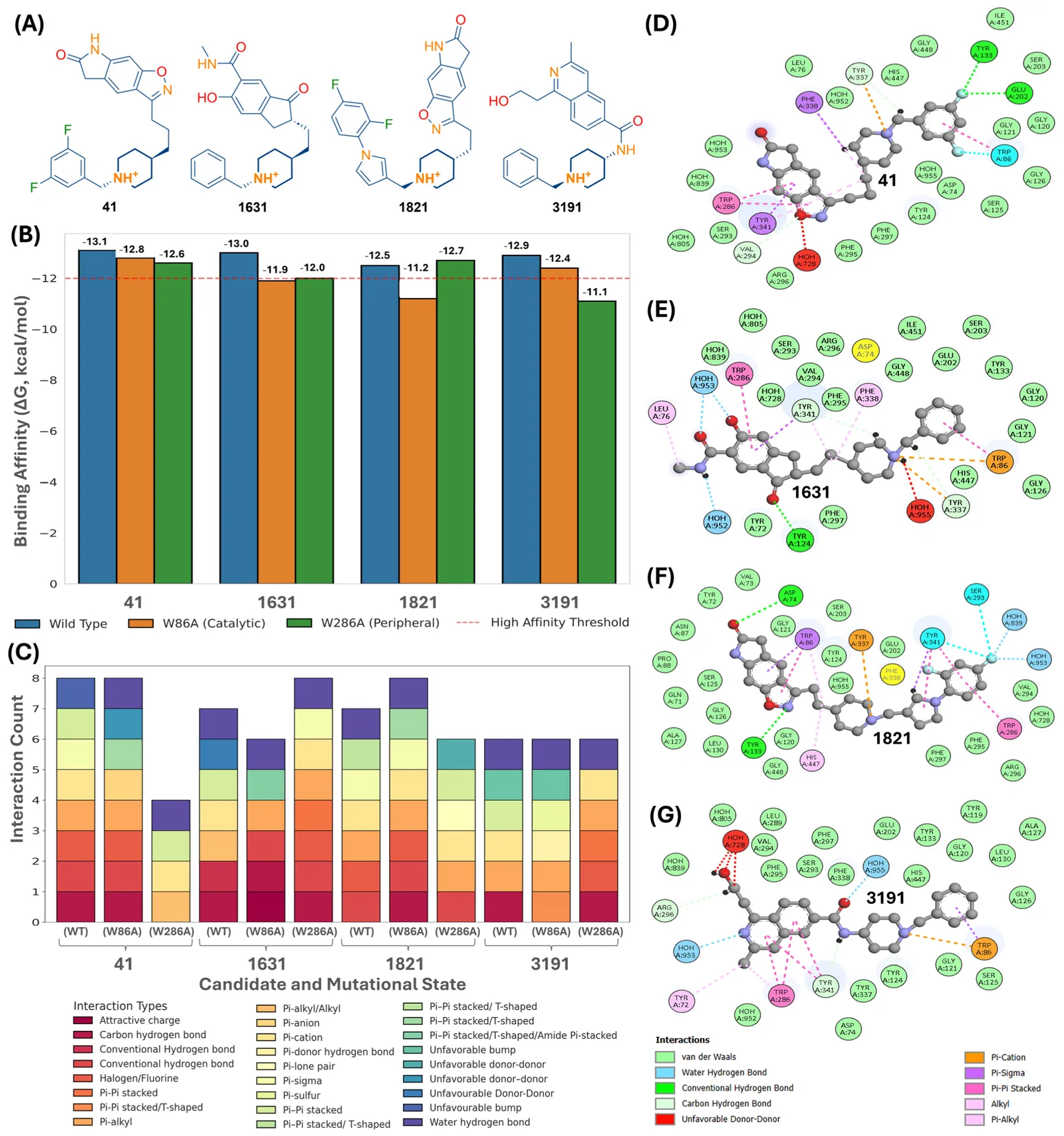
Topographical Screening and Binding Fingerprint Analysis. (**A**) Chemical scaffolds of the four prioritized probes: **41**, **1631**, **1821**, and **3191**. (**B**) Thermodynamic Penalty Map depicting binding free energies (ΔG in kcal/mol) across wild-type and mutated systems. (**C**) Interaction Distribution Analysis quantifying the shift in bonding modes (π-stacking, H-bonds) across the structural ensembles. (**D**–**G**) 2D interaction fingerprints of Probes **41**, **1631**, **1821**, and **3191**, respectively.

The structure-based ensemble molecular docking simulations executed across the native wild-type hAChE enzyme and its targeted, anchor-deficient variants provided a preliminary thermodynamic foundation for understanding the divergent molecular responses observed within the candidate pipeline. As quantified by the docking free energy profiles (Table 1), the four prioritized topographical probes exhibited robust, high-occupancy baseline affinities within the non-mutated enzyme gorge, satisfying the strict high-affinity threshold set at −12.0 kcal/mol (Figure 2B). However, upon the selective in silico knockout of the core catalytic base anchor (W86A) and the peripheral entrance rim gatekeeper (W286A), distinct variations in structural stability and mutational penalties (ΔΔG) emerged across the chemotypes (Table 1).

The structural robustness of the symmetrical bis-indanone Compound **1631** was shown to be anchored in its symmetrical, dual-site bridging architecture (Figure 2A), which achieved an optimal baseline wild-type affinity of −13.0 kcal/mol. Upon exposure to simulated mutational stress, Compound **1631** demonstrated remarkable stability, restricting its total thermodynamic penalties to a minor +1.1 kcal/mol within the catalytic W86A environment (yielding an affinity of −11.9 kcal/mol) and exactly +1.0 kcal/mol within the peripheral W286A locus (yielding an affinity of −12.0 kcal/mol).

Interaction distribution mapping confirmed that this resilience is a direct consequence of the ligand’s high chemical and spatial versatility within the channel (Figure 2C). While the single-point deletion of the primary Trp86 side chain effectively shatters the core cation–π binding pole at the catalytic active site base, the surrounding interface remains highly redundant. The atomistic fingerprint for Compound **1631** within the mutated environment (Figure 2E) reveals an instantaneous spatial redistribution of forces, characterized by the formation of localized water-mediated hydrogen bonds (HOH953) alongside directional, conventional hydrogen bonding with neighboring Tyr124.

Conversely, the deep-gorge penetrator probe, Ligand **1821** (Figure 2A), which lacks a balanced symmetrical topology, suffered a pronounced penalty of +1.3 kcal/mol under the identical W86A catalytic mutation, causing its affinity to collapse to −11.2 kcal/mol (Table 1). Atomistic fingerprinting of the Ligand **1821** ensemble (Figure 2F) indicates that its high baseline affinity depends almost entirely on rigid π-σ interactions anchored to the Trp86 side chain. Because its peripheral architecture cannot generate adequate compensatory networks when this anchor is removed, the compound experiences localized thermodynamic frustration and destabilization within the lower gorge matrix.

**Figure 3:**
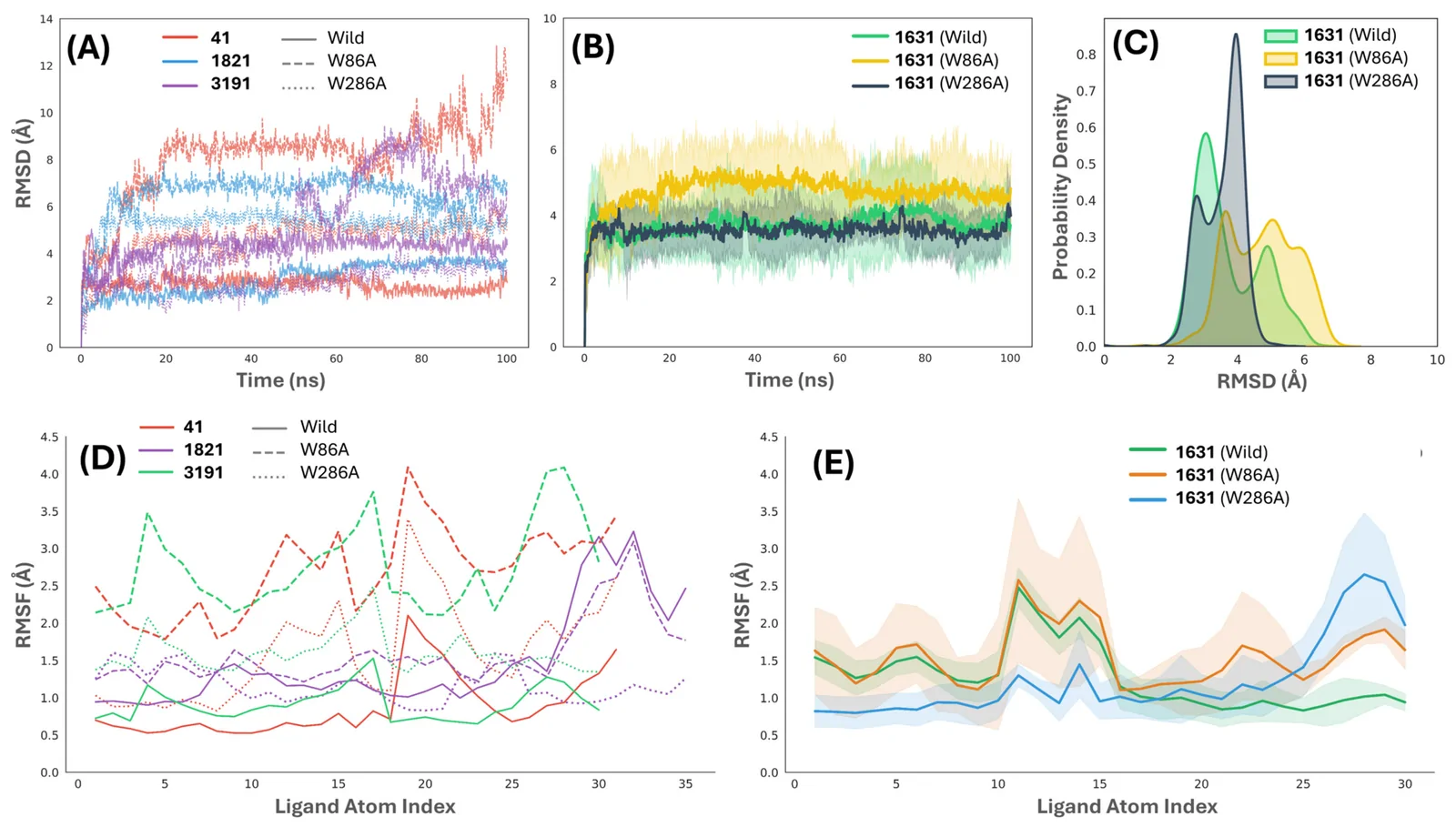
Continuous explicit-solvent molecular dynamics trajectories and atomistic fluctuation profiles for the hAChE topographical probes. (**A**) Heavy-atom RMSD profiles for reference probes **41**, **1821**, and **3191** across wild-type and mutated systems. (**B**) Multi-trajectory ligand RMSD ensembles for Lead **1631** across triplicate production runs (*n* = 3), with shaded bands tracking the Standard Error of the Mean (SEM). (**C**) Probability Density Function (PDF) curves mapping the statistical distribution and convergence of the localized RMSD populations for Lead **1631**. (**D**) Single-probe per-atom RMSF trajectories mapping single-atom deviations across the reference small molecules. (**E**) Ensemble atomic rigidity and per-atom fluctuation limits for Lead **1631** across the complete mutational matrix.

The non-canonical disruptor, Ligand **41** (Figure 3A), maintained stable docking scores within the standard operating error of the empirical scoring function, displaying minor variances of +0.3 kcal/mol and +0.5 kcal/mol across the W86A and W286A systems, respectively (Table 1). The interaction fingerprint for Ligand **41** within the catalytic mutant architecture (Figure 2D) confirms a successful static adaptive shift, marked by the establishment of an alternate sp^2^-anion interaction with residue Glu202 coupled with an extended, face-to-face π-π stacking matrix involving the side chains of Tyr341 and Tyr449.

Thermodynamic tracking across the structural ensembles (Figure 2C) indicates that the resilience of Ligand **41** is achieved through a significantly more constrained and narrower set of interaction classifications than the diverse, multi-pronged bridging mode seen in Compound **1631**.

This specialized dependency was further highlighted by evaluating the surface-targeted probe, Ligand **3191** (Figure 2A), which exhibited a severe binding affinity collapse, suffering a significant mutational penalty of +1.8 kcal/mol exclusively upon the deletion of the peripheral Trp286 residue (Table 1). The corresponding 2D interaction blueprint (Figure 2G) highlights that Ligand **3191** functions as a specialized cap that strictly occupies the peripheral anionic site. Because it lacks the molecular length required for deep-gorge penetration, the structural loss of its outer aromatic lid completely destabilizes its binding mode, leading to target disengagement.

### 2.3. Molecular Dynamics (MD) Simulations: Kinetic Screening and Stability

To evaluate the dynamic stability and structural resilience of the identified leads within the hAChE gorge, we transitioned from static docking models to 100 ns atomistic molecular dynamics (MD) simulations.

#### 2.3.1. Conformational Trajectories and Comparative Kinetic Screening (RMSD)

The time-dependent Root Mean Square Deviation (RMSD) of the ligand heavy atoms relative to the initial equilibrated coordinates served as the primary kinetic metric for separating structurally fragile architectures from truly resilient leads under active solvent pressure. Mapping the non-resilient reference chemotypes across the 100 ns production windows revealed severe kinetic vulnerabilities when exposed to single-point target mutations (Figure 3A). Specifically, Ligand **41** underwent a catastrophic unbinding event within the catalytic W86A mutant system. The heavy-atom RMSD profile of Ligand **41** rapidly escalated past 10 Å within the initial 50 ns of simulation time, signaling complete displacement from the catalytic active site base (Figure 3A). Statistical regression of this unbinding phase confirmed a lack of local equilibrium, yielding a high conformational drift slope of 0.042 Å/ns alongside an elevated late-stage Coefficient of Variation (CV = 20.35%). Similar kinetic instabilities were tracked for the reference probes Ligand **1821** and Ligand **3191**, which exhibited continuous, non-converging exploratory behavior and macro-conformational drift exceeding 5.0 Å in the absence of the Trp286 peripheral aromatic lid (Figure 3A).

In clear contrast to these volatile trajectories, Compound **1631** demonstrated outstanding structural stability across all tested conditions, validating its selection as a mutationally resilient architecture (Figure 3B). Replicated in independent triplicate trajectories to ensure statistical reproducibility, Compound **1631** achieved rapid equilibrium within the native wild-type enzyme gorge, settling at a tight, stable mean RMSD baseline of 3.72 ± 0.64 Å (Figure 3B). Crucially, when subjected to the catalytic anchor deletion (W86A), Compound **1631** avoided the structural unbinding seen in the reference compounds; instead, it executed a controlled, single-step spatial migration to an alternative stable plateau at 4.74 ± 0.63 Å (Figure 3B). The peripheral W286A knockout was accommodated with even greater structural tolerance, achieving a flattened, low-variance stabilized RMSD plateau at 3.54 ± 0.37 Å, mirroring the performance of the native wild-type trajectory (Figure 3B).

The reliability and thermodynamic relevance of these positional plateaus were supported by mathematical convergence metrics. Linear regression analysis modeling the final 50 ns production windows of Compound **1631** yielded near-zero drift slopes across all simulated backgrounds, including a wild-type trajectory slope of −0.0001 Å/ns and a peripheral mutant trajectory slope of −0.0016 Å/ns. The continuous probability density distribution curves (Figure 3C) clearly illustrated these tightly focused structural configurations, presenting uniform, sharp population frequencies at each respective free energy minimum. This systemic local convergence was further validated by temporal autocorrelation tracking across the production snapshots, which yielded an independent correlation factor as low as 0.02 within the W286A mutant matrix, confirming that the structural ensembles were statistically independent and uncorrelated prior to downstream analysis.

#### 2.3.2. Per-Atom Ligand Fluctuation and Ensemble Rigidity Mapping (RMSF)

To isolate the precise small-molecule functional groups driving either structural locking or conformational sliding within the 20 Å channel, the atomic trajectories were resolved at single-atom resolution using Root Mean Square Fluctuation (RMSF) profiling (Figure 3D,E). The non-symmetrical reference probes, which were optimized to target specific sub-pockets, exhibited severe mechanical destabilization and erratic atomic fluctuations immediately upon the deletion of their corresponding aromatic anchors (Figure 3D). The deep-gorge anchor, Ligand **41**, which relies tightly on rigid interactions with the catalytic floor, maintained a constrained wild-type atomic fluctuation baseline of 0.88 ± 0.12 Å (Figure 3D). Upon introduction of the W86A mutation, the absence of this stabilizing aromatic floor caused its internal fluctuations to rise sharply to a chaotic mean of 2.70 ± 0.44 Å, with its outer peripheral groups experiencing extreme deviations exceeding 3.2 Å (Figure 3D).

An identical mechanical collapse was tracked for the steric bulk probe, Ligand **3191**, whose mean per-atom fluctuation exploded from a stable wild-type baseline of 0.91 ± 0.15 Å to a highly volatile distribution of 2.74 ± 0.52 Å under catalytic W86A mutational stress (Figure 3D). This structural volatility highlights the vulnerability of traditional, single-pole steric blockade when challenged by single-point active-site variations. Conversely, the asymmetrical probe Ligand **1821** displayed a localized structural rigidification down to 1.17 ± 0.11 Å exclusively within the peripheral W286A knockout matrix, moving away from its elevated wild-type baseline of 1.48 ± 0.22 Å to accommodate peripheral space remodeling (Figure 3D).

In sharp contrast to the erratic fluctuations or narrow adaptations of the reference scaffolds, Compound **1631** maintained outstanding atomic rigidity across all simulated environments, as calculated from the independent triplicate trajectories (Figure 3E). In the native wild-type enzyme, the ensemble mean atomic fluctuation of the ligand settled at a highly controlled baseline of 1.27 ± 0.18 Å (Figure 3E). Crucially, during the spatial migration phase triggered by the catalytic W86A knockout, the ligand transitioned to its alternate energetic basin without experiencing disordered rotation or structural wobbling; its global per-atom fluctuation profile rose only fractionally to a stable mean of 1.56 ± 0.21 Å, preserving high geometric precision across its active functional groups (Figure 3E).

Furthermore, under the peripheral W286A knockout condition, Compound **1631** achieved an even tighter mechanical lock than in the native state, anchoring its symmetric core firmly at an impeccable fluctuation baseline of 1.23 ± 0.14 Å (Figure 3E). This low-variance atomic profile provides direct evidence that the symmetrical bridging architecture of Compound **1631** acts as a structural cross-shield. When one pocket-specific anchoring domain is compromised by an evolutionary mutation, the opposing, intact symmetrical wing holds the core structure firmly in place within the channel, proving that allosteric flexibility can be successfully leveraged to engineer deep-gorge neurotherapeutics with profound mutational resilience.

### 2.4. Post-MD Ensemble Validation: Conformational Landscapes and Structural Integrity

To bridge the gap between kinetic stability and thermodynamic equilibrium, we analyzed the conformational ensembles of Compound **1631** using FEL profiling and Ramachandran backbone dihedral distributions.

#### 2.4.1. Free Energy Landscape (FEL) Analysis: Topology of the Resilience Basin

**Figure 4:**
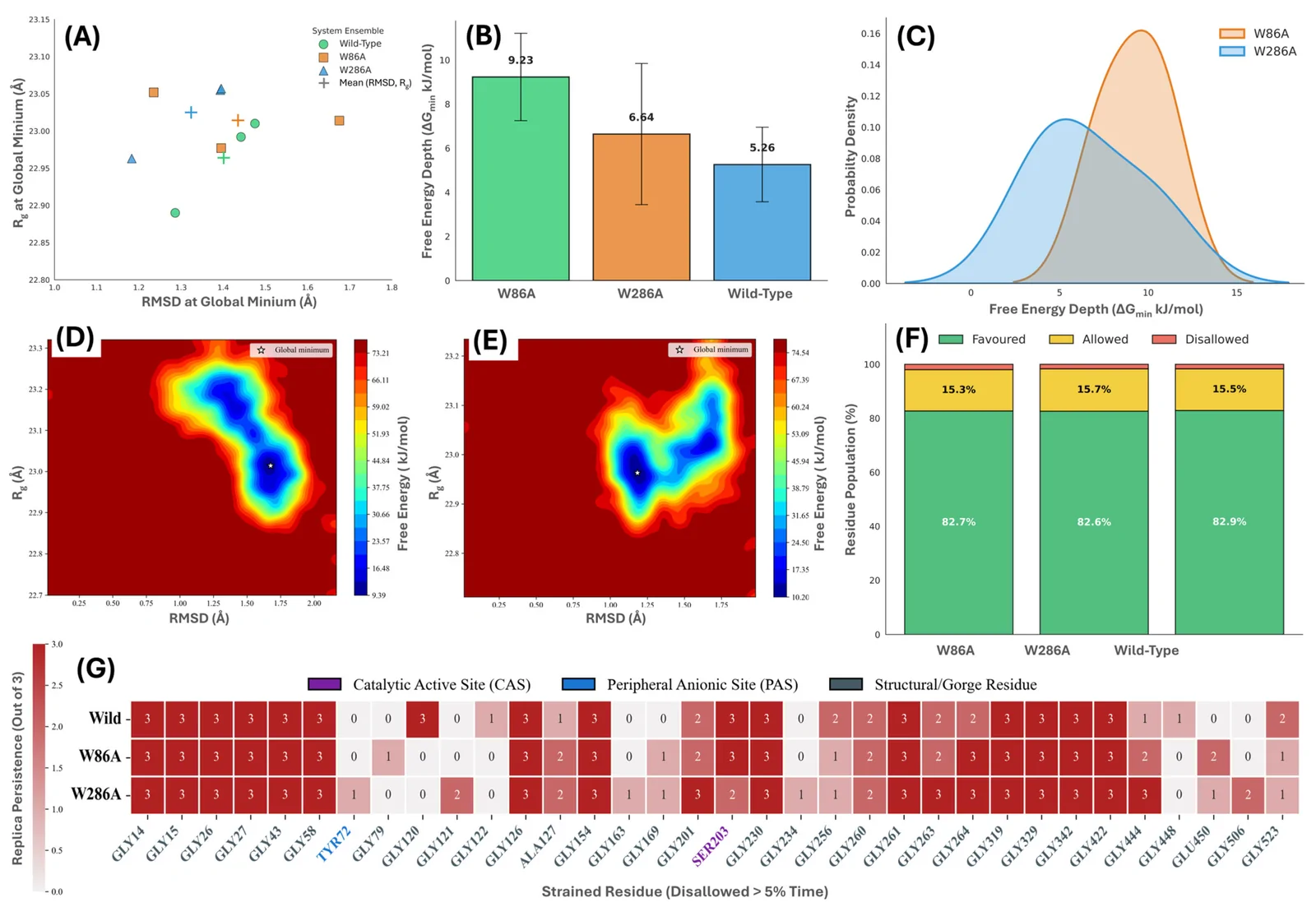
Post-simulation thermodynamic landscapes and macromolecular backbone validation profiles for Lead **1631**. (**A**) Discrete distribution and centroid clustering of independent trajectory minima projected onto ligand RMSD and protein R_g_ coordinate space. (**B**) Comparative bar chart tracking the absolute free energy depth (ΔG_min_ in kJ/mol) across the simulated backgrounds. (**C**) Continuous probability density function curves mapping population frequencies as a function of landscape energy depths. (**D**,**E**) Representative two-dimensional Gibbs FEL mapping the convergence funnels for the mutated ensembles. (**F**) Ramachandran dihedral distribution plots tracking enzyme secondary structure conservation. (**G**) Replica matrix mapping the temporal persistence of structural strain across disallowed active-site and gorge loop residues.

Projecting the underlying Gibbs free energy (ΔG) onto the orthogonal collective variables of ligand heavy-atom RMSD and protein radius of gyration (R_g_) mapped the thermodynamic convergence of the complexes. The tight clustering of the global energy minima centroids calculated across the independent triplicate trajectories (Figure 4A) confirmed that the explicit-solvent simulations successfully converged toward highly reproducible, discrete conformational states. Within the native wild-type ensemble, Compound **1631** occupied a well-defined, broad thermodynamic basin featuring a relative minimum depth (ΔG_min_) of 5.26 ± 1.69 kJ/mol (Figure 4B). However, the targeted knockout of the deep catalytic floor anchor (W86A) forced a profound restructuring of the underlying energy landscape. As visualized in the two-dimensional topological FEL projection (Figure 4D), the W86A mutant system re-equilibrated into a significantly deeper and more restricted energy funnel, reaching an absolute ΔG_min_ depth of 9.23 ± 1.98 kJ/mol (Figure 4B).

This clear ~4 kJ/mol increase in thermodynamic basin depth relative to the native state confirms that the alternative Solvation Rescue pose, which physically re-centers the core scaffold toward an increased ligand RMSD of 1.44 Å while preserving a stable, compact protein R_g_ of 23.01 Å, is thermodynamically superior to the original catalytic lock configuration.

The continuous probability density distribution curves (Figure 4C) further illustrate this Thermodynamic Locking effect. While the native wild-type population curve spans a broader coordinate landscape, confirming a higher degree of localized conformational breathing under baseline conditions, the mutated W86A and W286A systems (Figure 4E) collapse into remarkably sharp, concentrated probability distribution peaks centered at deeper energy wells. This thermodynamic profile demonstrates that Compound **1631** does not merely tolerate target-site perturbations passively. Instead, the symmetric chemotype actively re-optimizes its non-covalent network within the channel, driving the complex into a highly stable, low-entropy energy basin that prevents the structural detachment and unbinding observed in non-symmetrical reference probes.

#### 2.4.2. Macromolecular Secondary Structure Validation and Temporal Residue Stresses

To verify that the deep energy basins resolved in the free energy landscapes represented genuine ligand adaptations rather than artifacts of localized protein denaturation or unfolding, the time-dependent secondary structure integrity of the enzyme was evaluated. Continuous mapping of the peptide backbone dihedral angles (ϕ, ψ) across the 100 ns simulation ensembles (Figure 4F) confirmed that the core architecture of the hAChE matrix remained intact across all mutational backgrounds. Stereochemical distribution profiling showed that more than 82.6% of the total residue population resided comfortably within the strictly Favoured regions, while over 98% of the total ensemble coordinates clustered inside the combined Favoured and Allowed zones of the landscape (Figure 4F).

To further stress-test the structural health of the enzyme interface, a temporal Residue Persistence Analysis was executed on the minor fractional population tracking within the Disallowed regions of the landscape (Figure 4G). Unlike random thermal fluctuations, which would manifest as transient, inconsistent outliers across the replica matrix, the disallowed residues detected in these ensembles, specifically localized within structurally constrained loops containing glycine and proline turns, including Gly14, Gly126, and the catalytic Ser203, exhibited high temporal persistence across all three independent runs (Figure 4G).

The continuous preservation of the catalytic residue Ser203 in a highly strained, technically disallowed dihedral orientation is an established structural signature of the active AChE catalytic triad geometry. The precise duplication of this high-strain configuration across the trajectories proves that the OPLS4 force field parameterization accurately reproduced the electrostatic and steric conditions required for native enzymatic machinery. This structural stability under active mutational migration provides robust evidence that the resilience of Compound **1631** is achieved within a natively folded, functional macromolecular framework.

### 2.5. Comparative Metadynamics and the Thermodynamic Basis of Resilience

To definitively resolve the thermodynamic landscape of the primary lead, Compound **1631**, 150 ns of all-atom enhanced-sampling Metadynamics was performed across the wild-type (WT) and mutated enzyme systems.

#### 2.5.1. Native Wild-Type Equilibrium and Synchronized Dual-Site Anchoring

**Figure 5:**
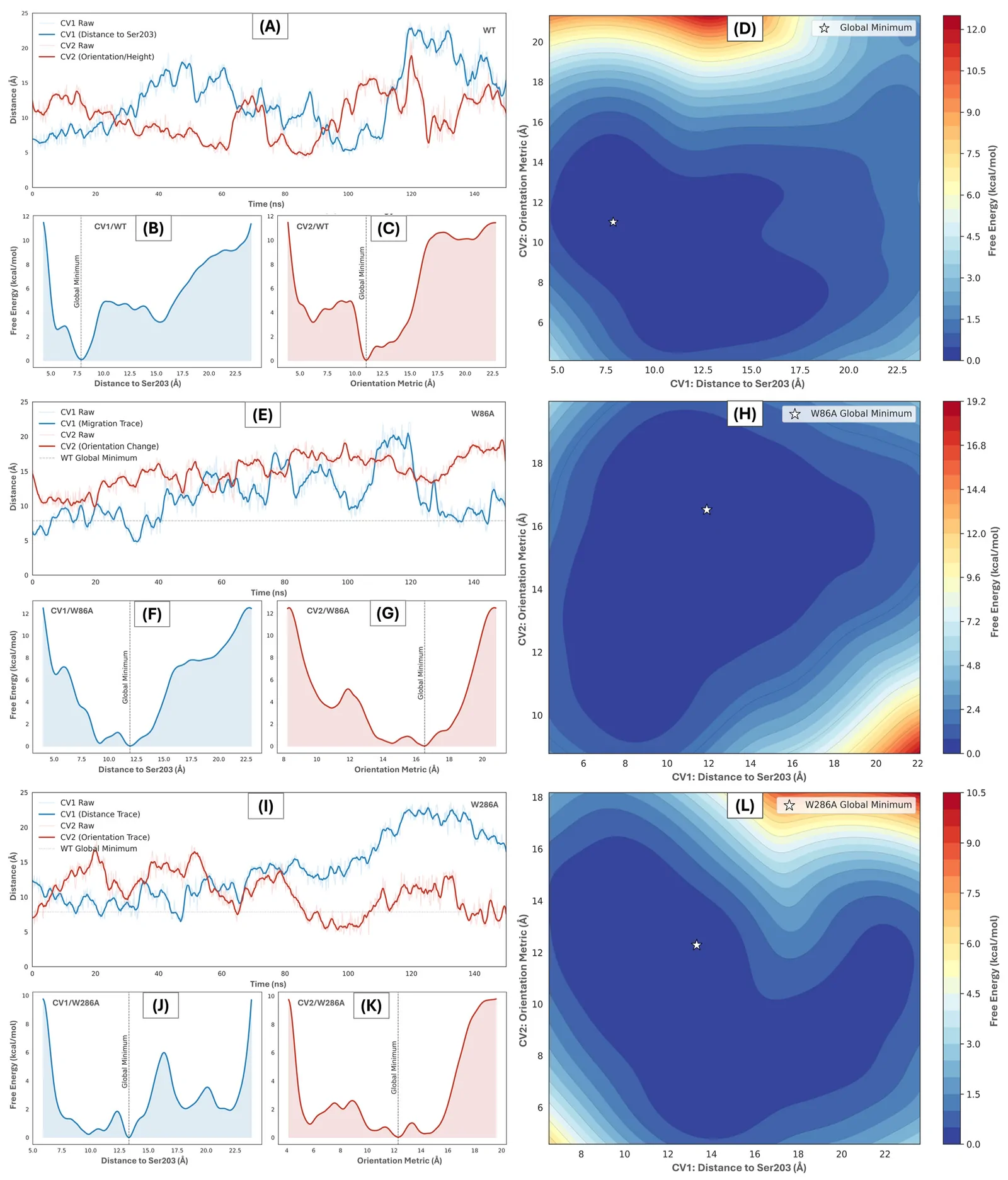
Enhanced-sampling Metadynamics trajectories and free energy topographies for Lead **1631** across hAChE variants. Panels (**A**–**D**) map the native wild-type system; (**E**–**H**) map the catalytic W86A mutant; and (**I**–**L**) map the peripheral W286A mutant. Columns from left to right track: time-dependent collective variable (CV) evolution over the 150 ns window; one-dimensional Free Energy Surfaces (FES) for CV1; one-dimensional FES for CV2; and two-dimensional FEL capturing the convergence and coordinate location of the absolute global minima (indicated via white stars).

To replace static empirical docking scoring functions with an explicit physical chemistry framework, 150 ns of all-atom enhanced-sampling Metadynamics was analyzed. This reconstruction of the underlying energy barriers confirmed that the native wild-type hAChE–**1631** complex populates a highly stable, high-occupancy resting state characterized by efficient phase-space exploration and structured breathing modes throughout the trajectory (Figure 5A). Quantitative profiling resolved a substantial absolute escape barrier of 11.37 kcal/mol governing the system (Table 2), indicating long-term complex stability and a prolonged target residence time prior to dissociation into bulk solvent.

Tracking the one-dimensional Free Energy Surfaces (FES) across the individual collective variables explicitly mapped the multi-site mechanics of the gorge. Along the Collective Variable 1 (CV1) projection, which tracks the deep-gorge distance between the protonated nitrogen moiety and the Trp86 Cα coordinate, the system settled into a localized global minimum centered at 7.87 Å (Figure 5B). This thermodynamic position was heavily reinforced by high internal transition barriers reaching approximately 4.92 kcal/mol at an outward coordinate distance of 10.24 Å (Figure 5B). These sharp energetic speed bumps prove that the ligand is rigidly held in its primary pose, requiring substantial thermal energy to transition into metastable intermediate states.

Concurrently, the one-dimensional FES modeling Collective Variable 2 (CV2), measuring the vertical height of the outer indanone core relative to the peripheral Trp286 Cα frame, identified a single, deep thermodynamic well at 11.02 Å guarded by a symmetric internal transition barrier of 4.96 kcal/mol (Figure 5C). Projection of these metrics into the unified two-dimensional Free Energy Landscape (Figure 5D) revealed a singular, highly focused energy funnel strictly enclosed within the low-energy contours. This thermodynamic topology confirms that the symmetrical bridge architecture of Compound **1631** occupies a low-entropy structural state, with its positively charged center anchored tightly at the CAS base while its hydrophobic core maintains synchronized engagement with the outer PAS.

#### 2.5.2. Catalytic Anchor Knockout: Solvation Rescue and the Broadened Metastable Well

Upon exposure to the catalytic active site knockout (W86A), the system underwent a major thermodynamic re-equilibration. The continuous collective variable evolution trajectory (Figure 5E) captured a controlled upward migration of the small molecule, abandoning its baseline coordinates near the active floor to reposition within the upper channel. While the loss of the primary aromatic side chain eliminated the sharp internal transition barriers seen in the native enzyme, reducing the internal speed bumps to a minor contour depth of <1.3 kcal/mol (Figure 5F,G), the compound remained securely trapped within the narrow walls of the gorge.

Evaluating the adjusted CV1 coordinate distance to the mutated Ala86 residue revealed a shallower, highly fluid profile, quantifying the loss of the canonical cation–π binding pole (Figure 5F). However, the parallel CV2 metric confirmed that the outer indanone core remained effectively tethered to the peripheral entry rim (Figure 5G), preventing target dissociation.

The resulting 1D FES for CV1 showed a definitive outward shift in the global minimum to 11.90 Å (Figure 5F), while the vertical orientation metric re-stabilized at an extended distance of 16.52 Å (Figure 5G). Two-dimensional topological landscape analysis (Figure 5H) confirmed the formation of a single, highly stable alternative thermodynamic basin. This structural shift provides direct evidence for the Solvation Rescue mechanism, where Compound **1631** accommodates the localized deletion of Trp86 by expanding its outer solvation shell and transitioning into a broadened, dynamically flexible configuration.

Remarkably, despite the increased internal volatility and the removal of the tight catalytic lock, the intact PAS anchor provides highly efficient compensatory tethering. This mechanism restricts the tumbling of the ligand and locks the system into its highest absolute escape barrier, reaching 12.54 kcal/mol (Table 2). This proves that deleting the core catalytic anchor does not disrupt or destabilize the binding pose. Instead, the available binding territory broadens, allowing the symmetric molecule to expand its non-bonded network while remaining securely confined within the gorge entrance, stabilized by favorable solvation interactions and secondary contacts.

#### 2.5.3. Peripheral Site Knockout: Thermodynamic Collapse and Mid-Gorge Fracturing

Evaluating the peripheral cap knockout (W286A) revealed a complete breakdown in dual-site energetic cooperativity, highlighting the ligand’s strict dependence on surface-level stacking networks. The time-dependent evolution trajectory (Figure 5I) confirmed that while Compound **1631** remained within the channel, its kinetic profile experienced an extreme increase in positional volatility. Trajectory analysis indicated that the outer indanone core engaged in erratic searching behaviors, exploring extreme solvent extensions up to 22.5 Å while simultaneously forcing the core scaffold to crash into the lower gorge interior at compressed vertical heights where CV2 < 6 Å (Figure 5I).

Evaluating the adjusted CV2 metric relative to the mutated Ala286 coordinate showed heightened volatility across the entrance rim (Figure 5K), which represents the structural disruption of the outer stacking network. Crucially, the CV1 metric confirmed that without the stabilization provided by the peripheral lid, the protonated nitrogen could not preserve its target coordinates with Trp86 deep within the active base. The entire small-molecule architecture was pulled upward, forcing the CV1 global minimum to shift outward to a distant coordinate of 13.32 Å (Figure 5J).

This displacement forced the ligand to settle into a floating, sub-optimal mid-gorge buffer zone. Unlike the singular funnels tracking in previous configurations, the 2D FEL for the peripheral W286A mutant (Figure 5L) split into a fractured topography featuring three distinct, disconnected energy islands. This topology indicates that the indanone core is tumbling through multiple separate, low-affinity orientations within the channel.

Concurrently, the absolute escape barrier collapsed to 9.69 kcal/mol (Table 2), marking the lowest values tracked across the study. This thermodynamic drop demonstrates that without the outer PAS lid, the lower CAS anchor cannot preserve its structural integrity. The dual-site engagement of Compound **1631** is strictly cooperative; removing the peripheral side chain shatters the thermodynamic landscape and stretches the small-molecule core, identifying the outer aromatic gatekeeper as a prerequisite for deep-gorge stability.

### 2.6. Binding Free Energy Analysis and the Energetics of Mutational Resilience

To quantitatively resolve the thermodynamic basis for the observed resilience of Ligand **1631**, we performed Molecular Mechanics/Generalized Born Surface Area (MM/GBSA) binding free energy calculations across triplicate trajectories. This analysis provides a high-fidelity decomposition of the forces, van der Waals, electrostatic, and desolvation, that allow the lead to maintain target occupancy despite catastrophic mutations to the gorge anchors (Figure 5).

#### 2.6.1. Global Thermodynamic Stability and Mutational Neutrality

**Figure 6:**
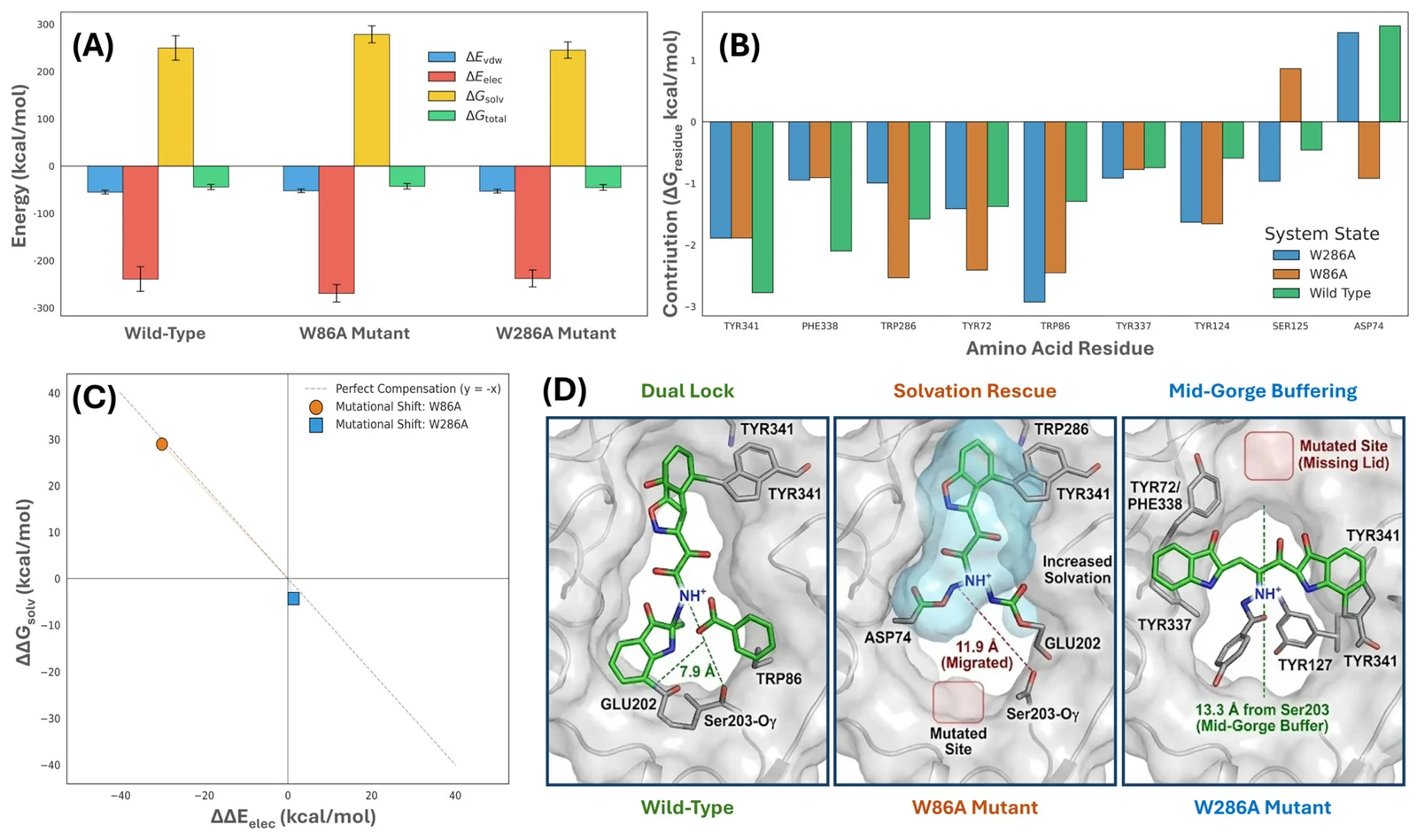
Binding Energetics and Thermodynamic Resilience of Ligand **1631**. (**A**) MM/GBSA component breakdown across triplicate trajectories, illustrating the electrostatic-solvation compensation in mutants. (**B**) Per-residue decomposition identifying key stabilizers (TYR341, PHE338) across wild-type and mutated systems. (**C**) Thermodynamic shift correlation; points near the y = −x diagonal mathematically confirm solvation rescue. (**D**) Representative MD snapshots visualizing the transition from the WT dual-lock to the migrated W86A and W286A resilience modes.

To quantitatively resolve the exact driving forces governing the observed resilience of Lead **1631**, end-state Molecular Mechanics/Generalized Born Surface Area (MM/GBSA) binding free energy calculations were executed across the independent triplicate trajectory pools. This structural decomposition isolated the specific contributions of van der Waals, electrostatic, and desolvation energies that allow the symmetrical lead to preserve target occupancy despite the loss of core gorge anchors (Figure 6A). The total calculated binding free energy (ΔG_total_) remained remarkably consistent across the native wild-type and mutated systems, providing definitive quantitative proof of thermodynamic resilience (Figure 6A). Within the non-mutated enzyme environment, Lead **1631** achieved a robust baseline binding affinity of −44.27 ± 5.53 kcal/mol (Table 3), driven by well-balanced, high-occupancy dual-site anchoring.

Crucially, upon the selective knockout of the deep catalytic floor anchor (W86A), the absolute binding free energy remained statistically unchanged at −42.68 ± 5.81 kcal/mol (Table 3), representing a negligible mutational penalty (ΔΔG_total_) of only +1.59 kcal/mol.

Most notably, the peripheral knockout mutation (W286A) resulted in a minor thermodynamic gain, reaching an absolute binding affinity of −45.20 ± 6.21 kcal/mol (Table 3). This complete absence of a significant ΔΔG_total_ penalty, despite losing the outer tryptophan side chain, demonstrates that the symmetrical architecture of Lead **1631** is optimized for mutational neutrality. This energetic profile confirms that the lead maintains a stable target residence time by dynamically redistributing its interaction forces, effectively surviving the structural loss of either primary binding pole without a collapse of complex stability.

#### 2.6.2. The Electrostatic-Solvation “See-Saw” (Solvation Rescue)

While the net binding free energy remained stable across the mutational matrix, the underlying component decomposition revealed a highly dynamic internal re-balancing of forces. The W86A mutant serves as a clear biophysical example of the Solvation Rescue mechanism, where losing a specific structural side chain is compensated for by a complete redistribution of the ligand’s electronic footprint (Figure 6A). Quantitative shift analysis indicated that the deletion of the Trp86 anchor triggers a spatial migration of the core toward the upper gorge periphery (shifting from 7.87 Å to 11.90 Å), resulting in a massive electrostatic energy gain (ΔΔE_elec_) of −30.10 kcal/mol relative to the wild-type baseline (ΔΔE_elec_W86A_ = −269.23 ± 18.54 kcal/mol vs. ΔΔE_elec_WT_ = −293.13 ± 26.15 kcal/mol) (Table 3). This substantial shift indicates that the ligand re-orients within the channel to optimize alternative ionic interactions with accessible residues, such as Asp74 and Glu202, once freed from the steric constraints of the native Trp86 indole ring.

However, this dramatic gain in electrostatic stabilization was almost perfectly counterbalanced by an equivalent increase in the polar desolvation penalty (ΔΔG_solv_) of +28.98 kcal/mol (ΔG_solv_W86A_ = 278.85 ± 18.03 kcal/mol vs. ΔG_solv_WT_ = 249.87 ± 25.92 kcal/mol) (Table 3). In the thermodynamic shift correlation plot (Figure 6C), the catalytic W86A mutation maps directly onto the y = −x diagonal, mathematically confirming the existence of an active “solvation see-saw”. This phenomenon proves that Lead **1631** is not a static inhibitor; it functions as a dynamically adaptable lead that balances the enthalpic cost of desolvating its polar groups against the stabilization energy gained through novel electrostatic contacts. This compensatory mechanism effectively rescues the overall binding affinity, ensuring that the net energetic penalty for losing a core catalytic anchor is minimized to a minor +1.59 kcal/mol.

#### 2.6.3. Residue-Level Load Shifting and Compensatory Anchoring Networks

The per-residue free energy decomposition map isolated the specific amino acid residues responsible for maintaining the thermodynamic integrity of the complex when primary anchors were compromised (Figure 6B). In the native wild-type system, the binding energy was concentrated at the two primary poles of the gorge. Residues Tyr341 (−2.77 kcal/mol) and Phe338 (−2.10 kcal/mol) functioned as the dominant stabilizers (Figure 6B), locking the ligand in place alongside the native Trp86 and Trp286 side chains to distribute the workload across four distinct aromatic stacking centers.

When this structural equilibrium was disturbed by target mutations, the energy load shifted to secondary stabilizers. In the catalytic W86A mutant, losing the Trp86 contact did not trigger a loss of affinity because neighboring residues, specifically Tyr341 and Tyr72, increased their relative contributions (Figure 6B). This localized load shifting is a hallmark of structural resilience, proving that the protein–ligand interface possesses sufficient redundancy to prevent a total collapse of the binding network.

Similarly, within the peripheral W286A mutant environment, a Mid-Gorge Buffering effect was resolved. As the ligand re-centered itself within the channel to compensate for the missing peripheral lid, residue-level decomposition confirmed that Tyr337 and Phe338 became the primary stabilizing anchors (Figure 6B). Interestingly, acidic residues like Asp74, which typically present a desolvation penalty in the wild-type state, shifted toward less penalizing values or favorable electrostatic contacts in the mutated systems (Figure 6B). This transition from a Dual-Lock to a Compensatory Anchor model illustrates how Lead **1631** utilizes its symmetrical core to preserve near-zero mutational penalties across the heterogeneous landscape of the gorge.

#### 2.6.4. Structural Manifestations of Energetic Adaptation

The thermodynamic transitions and energetic re-balancing quantified across the ensembles were visually validated by the representative binding modes captured during the production timelines (Figure 6D). In the native wild-type system, Lead occupied a strictly defined Dual-Lock orientation, symmetrically distributed within the **1631** channel with its central protonated nitrogen localized at an optimal distance of 7.9 Å from the active Ser203-Oγ residue (Figure 6D). This structural baseline matches the high transition barriers tracked in the free energy surfaces, keeping the ligand anchored in a singular, low-entropy state under baseline conditions.

However, upon the loss of the Trp86 catalytic anchor, structural monitoring confirmed a spatial migration toward the peripheral site, settling into a Solvation Rescue pose localized at a mean distance of 11.9 Å from the active base (Figure 6D). In this state, the ligand abandons the deep gorge floor to establish novel, stabilizing electrostatic contacts with Asp74 and Glu202 (Figure 6D). The structural snapshots clearly illustrate the expansion of the localized solvation cloud around the core scaffold (Figure 6D), providing the physical medium for the compensatory tethering that preserves the global binding affinity.

Conversely, within the peripheral W286A mutant system, the ligand adopted a stable Mid-Gorge Buffer state, centering its protonated core at 13.3 Å from the catalytic base (Figure 6D). Despite the complete absence of the peripheral Trp286 lid, the structural symmetry of Lead **1631** allowed it to re-center its energy funnel within the gorge interior (Figure 6D). This positioning prevented scaffold detachment and maintained a robust target occupancy that explains the minimal mutational penalty observed in the total binding free energy.

### 2.7. Dynamic Cross-Correlation: Mechanical Rewiring and Allosteric Resilience

**Figure 7:**
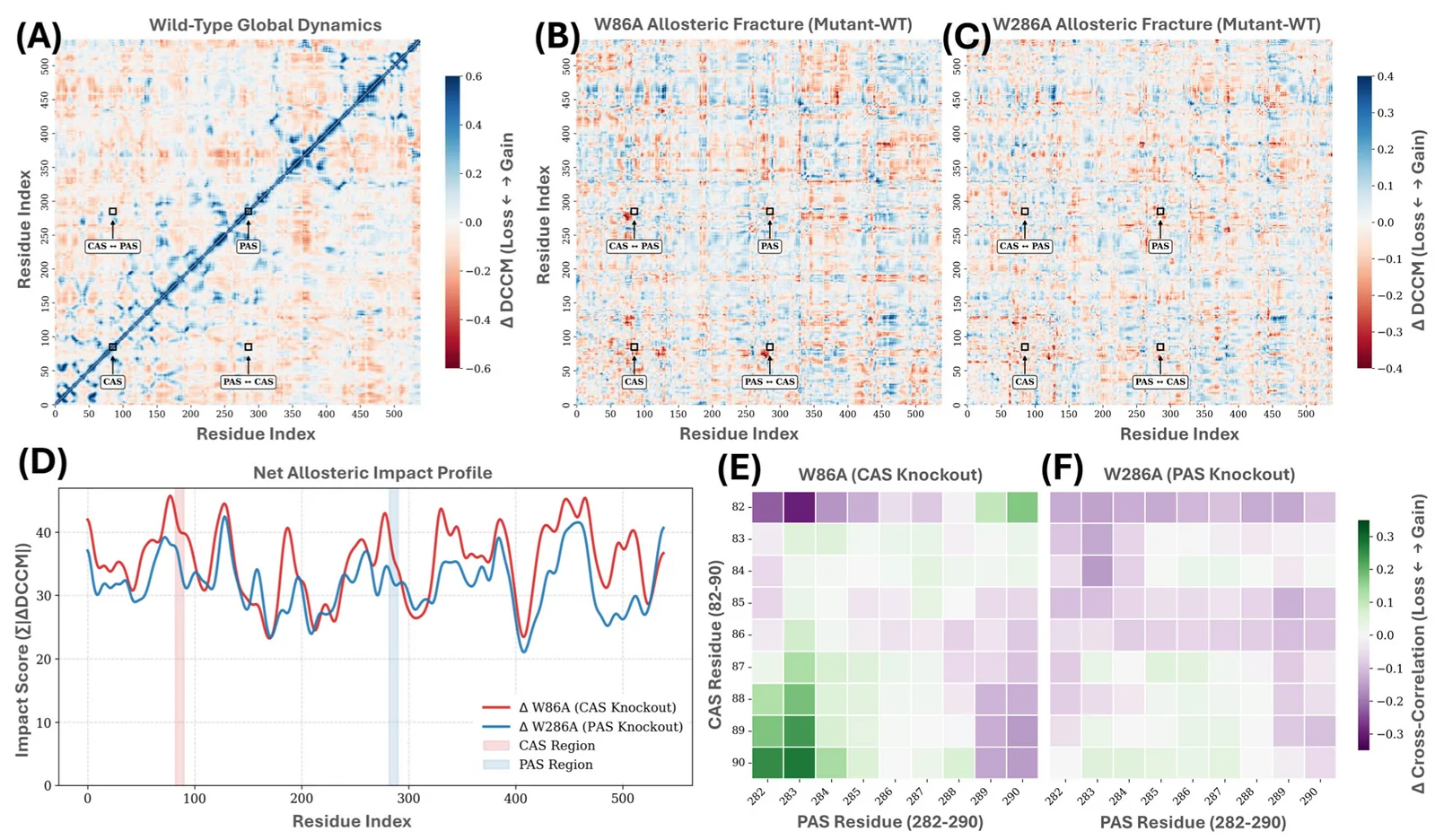
Dynamic Cross-Correlation and Allosteric Rewiring. (**A**) Wild-type global dynamics showing highly integrated CAS-PAS coupling. (**B**,**C**) Allosteric Fracture matrices (ΔDCCM) illustrating the vibrational decoupling induced by W86A and W286A mutations. (**D**) Net Allosteric Impact profile highlighting the residues most sensitive to mechanical rewiring. (**E**,**F**) Correlation profiles for the CAS and PAS Knockout states, showing the shift from synchronized to anti-correlated clamping motions.

The final component of this biophysical investigation mapped the Dynamic Cross-Correlation Matrices (DCCM) to resolve how selective mutational stress alters long-range mechanical communication and vibrational coupling networks within the hAChE gorge. By analyzing the correlated displacements of all 539 residue Cα positions across the independent triplicate simulation ensembles, a robust allosteric rewiring mechanism was identified underpinning the resilience of Lead **1631** (Figure 7).

The native wild-type hAChE–**1631** complex exhibited a highly integrated vibrational profile, characterized by strong positive correlation features running along the primary catalytic axis (Figure 7A). Within this unmutated baseline state, the Catalytic Anionic Site (CAS) and the Peripheral Anionic Site (PAS) regions displayed synchronized coupling with a distinct positive correlation coefficient of +0.0447 (Figure 7A). This baseline mechanical synchrony demonstrates that in the presence of the symmetrical bridging lead, the two functional ends of the 20 Å gorge vibrate as a single coherent structural unit, maintaining a stable dual-site lock on the small molecule under native conditions.

The introduction of the targeted W86A (CAS knockout) and W286A (PAS knockout) mutations induced a profound Allosteric Fracture within the core enzyme framework (Figure 7B,C). The computed cross-correlation difference matrices (ΔDCCM) demonstrated that these single-point side chain deletions do not merely cause localized structural damage near the mutation site. Instead, they decouple long-range mechanical communication between the gorge entrance rim and the active site floor, fracturing the unified vibrational pathways tracked in the native state.

Within the catalytic W86A mutant system (Figure 7E), the structural loss of the deep aromatic anchor triggered a clean inversion toward anti-correlated motion. The specific mechanical coupling between the CAS and PAS regions dropped down to a negative correlation value of −0.0355, representing a severe net structural fracture of −0.0802 relative to the wild-type baseline. This mechanical inversion implies that the opposite ends of the gorge now move in direct out-of-phase opposition to one another. This anti-correlation functions as an adaptive clamping mechanism, where the rigidified peripheral PAS matrix actively secures the ligand’s migrated Solvation Rescue pose while the vacant CAS floor retains higher structural flexibility to minimize localized stress. An identical mechanical decoupling and out-of-phase shift was captured within the peripheral W286A knockout matrix (Figure 7F), where the corresponding CAS–PAS coupling fell to a negative value of −0.0412.

The Net Allosteric Impact Profile (Figure 7D) mapped the global sensitivity of individual residues to this mutational stress, highlighting that the amino acids surrounding the 20 Å gorge channel (specifically residues 70–120 and 280–340) undergo the most significant mechanical rewiring shifts. Most notably, the peripheral W286A mutation induced a localized positive coupling spike between the catalytic nucleophile Ser203 and the mid-gorge bottleneck residues, quantifying an increase of +0.103 (Figure 7D).

This Mechanical Rescue confirms that the symmetric scaffold of Lead **1631** serves as a functional physical bridge within the channel. When the outer peripheral anchor is evolutionarily lost, the ligand actively transmits core vibrational signals directly down to the catalytic floor, reinforcing the geometry of the active site. This continuous re-optimization of internal vibrations, transitioning from synchronized fluctuations into a compensated mechanical clamping mode, provides direct proof that the structural resilience of Lead **1631** is a product of its ability to exploit and rewire the enzyme’s inherent allosteric plasticity.

## 3. Materials and Methods

### 3.1. Dataset Curation, Physicochemical Filtering, and Structural Standardization

To establish a statistically robust baseline for chemical space mapping and subsequent predictive modeling, a comprehensive dataset of small-molecule hAChE inhibitors was curated from the Papyrus database [28]. The collection was restricted to historical entries with experimentally validated, direct in vitro bioactivity data derived from uniform assay protocols measuring half-maximal inhibitory concentrations (IC_50_) or exact inhibition constants (K_i_). Converting raw experimental values into standardized, logarithmic pChEMBL units provided a broad, continuous bioactivity distribution suitable for training machine learning scoring functions without introducing classification bias.

To bias the training data and downstream generative design toward lead-like chemical space optimized for CNS targets, a multi-parameter physicochemical filtering protocol was systematically enforced. The molecular weight was restricted to a specific window to provide the spatial mass required to simultaneously span the 20 Å trajectory bridging the CAS and the PAS, while capping molecular bulk to prevent steric exclusion at the narrow gorge rim. Lipophilicity was optimized by enforcing a calculated partition coefficient (cLogP) window to maximize passive BBB permeation, thereby enhancing lipophilic ligand efficiency while mitigating the pharmacokinetic pitfalls of non-specific lipid sequestration and high metabolic clearance. Concurrently, the topological polar surface area (TPSA) was restricted to a strict window established for high-occupancy brain penetration. Thermodynamic desolvation penalties were minimized by restricting hydrogen bond donors (HBD) to eliminate unfavorable polar transitions across the lipid bilayer, while a designated range of hydrogen bond acceptors (HBA) was permitted to facilitate compensatory interaction networks within the enzyme interior. Finally, strict structural architecture constraints mandating a specific range of aromatic rings and exactly one basic nitrogen atom were enforced. This baseline composition guarantees that the prioritized compounds possess the electronic attributes necessary for intensive π-π stacking networks and maintain a protonated, positively charged state at physiological pH to enable long-range cation–π interactions with deep-gorge aromatic residues.

Following the property filters, a multi-step structural standardization was executed using the RDKit toolkit (version 2026.03.2) [29]. All structures were stripped of counterions, salts, and solvent fragments to ensure uniform molecular representation. Valence states were verified, explicit hydrogens were assigned, and stereochemical designations were standardized to generate clean canonical SMILES strings. Duplicate entries resulting from identical canonical representations were discarded. This curated and refined dataset served as the core training set for the Random Forest-based QSAR scoring functions and provided the chemical reference space for evaluating structural resilience within the human hAChE gorge.

### 3.2. QSAR Classification Modeling, Applicability Domain, and Topographical Diversity Triage

To effectively guide the deep learning de novo molecular generation process and guarantee the architectural reliability of the predicted bioactivities, a QSAR classification scoring function was established using a Random Forest ensemble architecture in DrugEx framework [30]. While deep generative frameworks are highly efficient at traversing chemical space, they remain fundamentally prone to objective-function exploitation and optimization gaming, frequently generating unrealistic molecular configurations or structural anomalies that yield artificially inflated predictive scores. To structurally safeguard the pipeline against these generative artifacts, a rigorous machine learning-based Applicability Domain (AD) filter was developed as an absolute gatekeeper for candidate prioritization. The boundary of the AD was mathematically defined using a K-Nearest Neighbors (k-NN) instance-based learning algorithm operating within an orthogonal high-dimensional bit space. Molecular structures were vectorized into dense Morgan molecular fingerprints utilizing a localized radius of 2 (r = 2, equivalent to circular ECFP4 topologies) compressed to a fixed bit-string length of 2048 bits [31]. Inter-molecular structural proximities within this fingerprint matrix were calculated utilizing an inverse Jaccard distance metric to quantify absolute topological dissimilarity relative to the reference hAChE training coordinates.

To establish a mathematically defensible and operationally strict classification boundary, the k-NN distance threshold was empirically calibrated utilizing an external validation cohort composed of an equal distribution of active reference ligands and distinct structural decoys. The active subset comprised verified hAChE inhibitors drawn directly from the curated database, whereas the decoy subset was constructed from structurally diverse, commercially available non-inhibitory small molecules spanning multiple distinct chemical classes, including native amino acids, simple organic solvents, non-steroidal anti-inflammatory drugs (NSAIDs), and organic vitamins. By evaluating the continuous Jaccard distance distributions between the active and decoy populations, an optimal hard Euclidean distance threshold was identified using a grid-search optimization protocol designed to maximize classification sensitivity for true positive inhibitors while maintaining absolute specificity to reject structural outliers.

Following the de novo generative phase directed by the integrated Random Forest scoring function and the successive application of the multi-parameter ADMET, shape index (I_shape_ > 0.60), and Pan-Assay Interference Compounds (PAINS) zero-tolerance filters, the surviving virtual library was subjected to a rigorous structural diversity triage. To eliminate localized structural redundancy, prevent analog biasing, and ensure the selection of unique chemotypes for downstream atomistic simulation, an automated clustering protocol was enforced. All qualified generated configurations were converted into independent 2048-bit Morgan fingerprints (r = 2) via the RDKit toolkit (version 2026.03.2), and a complete, symmetric pairwise Tanimoto similarity matrix was computed across the population. Structural clustering was executed using the non-hierarchical, leader-based Butina clustering algorithm parameterized with a stringent Tanimoto similarity threshold [32]. Any candidate molecule exhibiting a similarity coefficient exceeding this designated threshold relative to a cluster center was flagged as highly redundant and discarded. The systematic extraction of the true cluster centroids effectively condensed the virtual library down to a finalized ensemble of unique candidate chemotypes, guaranteeing an optimized, broad coverage of the newly explored chemical space prior to the implementation of structure-based molecular docking.

### 3.3. Structure-Based Ensemble Docking and Non-Covalent Interaction Profiling

To systematically evaluate the binding thermodynamics and structural complementarity of the prioritized virtual library within the constrained architecture of the hAChE gorge, a rigorous structure-based molecular docking protocol was implemented.

#### 3.3.1. Target Preparation and Grid Generation

Target preparation was initiated by retrieving the high-resolution crystal structure of human Acetylcholinesterase from the Protein Data Bank utilizing the structural coordinates of PDB ID: 4EY7 [33,34]. The macromolecular structure was processed and prepared within the MZDock computational suite (version 2026.1.0). Receptor preparation involved the removal of all crystallographic water molecules, non-receptor co-factors, and heteroatoms, followed by the addition of polar hydrogen atoms and the assignment of partial charges utilizing the Merck Molecular Force Field 94 (MMFF94) to ensure proper electrostatic representation [34]. Ligand geometries were concurrently optimized and minimized using the identical MMFF94 force field parametrization to maintain rigorous energetic and thermodynamic consistency across the virtual library. To simulate mutational stress and evaluate resistance to target-site variations, in silico site-directed mutagenesis was performed to generate the homogeneous W86A (CAS knockout) and W286A (PAS knockout) enzyme variants utilizing the SWISS-PDB Viewer toolkit (version 4.1.0) [35]. Rather than using arbitrary or static coordinate boxes, the docking search space was dynamically restricted by constructing a definitive grid box centered around the geometric centroid of the co-crystallized donepezil ligand. A 6 Å buffer space was applied in all three spatial dimensions, completely encompassing the catalytic base, the mid-gorge bottleneck, and the peripheral entrance rim, thereby ensuring unconstrained structural exploration within the entire 20 Å enzyme gorge.

#### 3.3.2. Docking Protocol and Empirical Scoring

The virtual screening simulations were executed using the Smina engine integrated within the MZDock platform, driven by an empirical scoring function optimized for robust binding free energy (ΔG) predictions [36,37]. This setup utilized an exhaustive stochastic memetic algorithm to ensure thorough sampling of the ligand’s conformational space within the deep, narrow gorge channel. Throughout the initial screening phase, the receptor backbone and side chains remained rigid, while the candidate ligands were treated as fully flexible, allowing all rotatable bonds to dynamically adapt to the spatial constraints of the channel. The resulting binding configurations were ranked according to their binding affinities to serve as the baseline thermodynamic profile for the initial lead prioritization phase.

#### 3.3.3. Automated Interaction Fingerprinting

Following pose ranking, the top-tier protein–ligand complexes were subjected to automated non-covalent interaction fingerprinting to map the precise networks driving target engagement. Spatial interaction networks were resolved using the Protein–Ligand Interaction Profiler (PLIP, version 2.3.0) and verified via the Discovery Studio Visualizer (version 2025.1.0) [38,39]. The geometric search criteria were parameterized to identify and quantify three vital contact vectors across the mutated ensembles. Parallel and perpendicular face-to-face π-stacking networks were resolved to monitor contact retention with core aromatic residues, specifically targeting interactions with Trp86, Tyr337, and Trp286. Directional hydrogen bonding topologies were mapped to evaluate electrostatic stability relative to the core catalytic triad, specifically monitoring donor-acceptor distances and angle alignments with Ser203, His447, and Glu334. Concurrently, steric and hydrophobic contacts were mapped to resolve van der Waals complementarity and lipophilic surface alignment within the hydrophobic barrier region of the mid-gorge channel. The spatial frequencies and classifications of these resolved contacts were systematically aggregated, providing a quantitative structural basis for discarding dynamically fragile configurations and selecting the most balanced chemotypes for subsequent all-atom molecular dynamics stress tests.

### 3.4. Molecular Dynamics (MD) Simulations and Statistical Robustness

To evaluate the dynamic stability, conformational adaptability, and structural resilience of the prioritized lead chemotypes under physiological conditions, all-atom MD simulations were executed.

#### 3.4.1. Solvation Environment Setup and Multi-Stage Relaxation

All system preparation and network building procedures were conducted using the System Builder utility within the Desmond Molecular Dynamics System (Schrödinger Release 2023-1) [40]. The top-ranked protein–ligand configurations derived from the topographical docking phase were imported and centered within an orthorhombic simulation volume. The boundary of the box was parameterized to maintain a minimum buffer distance of 10 Å from the outermost atoms of the protein surface into the bulk solvent. The explicit aqueous environment was modeled using the Simple Point Charge (SPC) model [41]. To approximate standard physiological conditions, the solvated systems were neutralized through the targeted addition of sodium and chloride counterions, adjusting the net background salt concentration to an ionic strength of 0.15 M NaCl. Macromolecular structures and small-molecule topologies were parameterized using the high-accuracy OPLS4 force field [42,43], optimizing the representation of non-bonded electrostatics, van der Waals interactions, and torsional potentials across the protein–ligand interfaces. Prior to initiating unrestrained production simulations, each system was subjected to the default Desmond six-stage relaxation protocol to eliminate localized steric clashes and electronic strain. This pre-equilibration workflow applied localized, descending harmonic restraints to the solute heavy atoms while systematically alternating between Isochoric–Isothermal (NVT) and Isobaric–Isothermal (NPT) ensembles, safely relaxing the solvent environment and stabilizing the system at 310.15 K and 1 bar.

#### 3.4.2. Production Trajectories and High-Performance Integration

Unrestrained production MD simulations were executed for an aggregate duration of 100 ns for each primary ligand–receptor complex. To ensure strict statistical rigor, eliminate stochastic artifacts, and validate the reproducibility of the observed trajectories, the lead compound was simulated in independent triplicate production runs (*n* = 3) across the native wild-type enzyme, the CAS-localized W86A variant, and the PAS-localized W286A variant. The remaining topographical reference probes were subjected to single-trajectory 100 ns kinetic stress tests to establish comparative baseline behavior. Long-range electrostatic interactions were resolved with high spatial accuracy using the Particle Mesh Ewald (PME) method [44], parameterized with a grid spacing threshold of 0.8 Å. All covalent bonds involving hydrogen atoms were rigidly constrained using the M-SHAKE algorithm [45], which eliminated high-frequency vibrational degrees of freedom and permitted the safe deployment of a 2 fs integration timestep within a reference RESPA integrator scheme.

#### 3.4.3. Trajectory Processing and Statistical Equilibrium Metrics

Post-simulation trajectory analysis and processing for both standard molecular dynamics and metadynamics were executed utilizing the Simulation Interaction Diagram (SID) tool integrated within the Maestro graphical interface (Schrodinger, LLC) [46,47]. Geometric tracking metrics, including the Root Mean Square Deviation (RMSD) of the protein backbone and ligand heavy atoms, were monitored continuously relative to the initial equilibrated coordinates to quantify macro-structural deviations. Localized residue fluctuations were resolved by calculating the Root Mean Square Fluctuation (RMSF) of individual alpha-carbon positions to isolate regions of structural rigidification or destabilization. Global structural compactness and solvent-shielding efficiency were monitored throughout by calculating the time-dependent Radius of Gyration (Rg) and the Solvent Accessible Surface Area (SASA) of the protein matrix.

#### 3.4.4. Dynamic Cross-Correlation Matrix (DCCM) Mechanics

To systematically resolve the long-range allosteric communication networks and vibrational coupling behaviors within the hAChE gorge, Dynamic Cross-Correlation Matrices (DCCM) were constructed based on the spatial displacement vectors of all alpha-carbon backbone atoms across the simulation ensembles [48,49]. The trajectory coordinates were pre-aligned to remove rotational and translational artifacts utilizing MDAnalysis, and the cross-correlation of atomic fluctuations was mathematically resolved utilizing the NumPy library [50]. The dimensionless cross-correlation coefficient, *C_ij_*, which quantifies the degree of synchronized motion between any two given residues *i* and *j*, was computed utilizing the formal statistical mechanical definition:

$$C_{ij}=\frac{\left\langle ∆r_{i}\cdot ∆r_{j}\right\rangle }{\left\langle ∆r_{i}^{2}\rangle \langle ∆r_{j}^{2}\right\rangle }$$

where Δ*r_i_* is the displacement of atom *i* from its mean position. Difference matrices (ΔDCCM) were computed by subtracting the wild-type correlation values from the mutant matrices to isolate the mechanical Allosteric Fractures induced by mutational stress. where Δ*r_i_* and Δ*r_j_* represent the instantaneous displacement vectors of atoms *i* and *j* from their respective time-averaged mean positions over the production trajectory, and the angular brackets denote the ensemble average over the analyzed snapshots. The resulting coefficients were organized into a symmetric matrix bounded by values ranging from −1.0 (perfectly anti-correlated, out-of-phase motion) to +1.0 (perfectly correlated, in-phase synchronized motion). To isolate the specific mechanical shifts and mechanical rewiring events triggered by mutational stress, spatial difference matrices (ΔDCCM) were constructed by directly subtracting the native wild-type correlation coefficients from the corresponding mutant matrix fields (DCCM_mutant_–DCCM_WT_). This subtraction isolated the precise allosteric fractures and compensatory clamping modes induced across the enzyme structure.

### 3.5. Post MD Analysis

#### 3.5.1. Free Energy Landscape (FEL) Construction

To resolve the long-term thermodynamic stability and identify the low-energy conformational states of the protein–ligand complex, two-dimensional FEL were constructed [51]. To ensure comprehensive sampling of the conformational phase space and minimize stochastic variations, the independent triplicate production trajectories for lead compound were temporally concatenated prior to analysis. The landscapes were generated by projecting the underlying Gibbs free energy (ΔG) onto two orthogonal collective variables chosen as principal reaction coordinates: the ligand heavy-atom RMSD, which monitors structural orientation relative to the initial binding pose, and the protein R_g_, which evaluates global macromolecular compactness. The continuous phase space was discretized into a two-dimensional probability density matrix. To eliminate localized statistical noise arising from thermal fluctuations and to resolve clear thermodynamic basins, the raw energy matrices were subjected to localized Gaussian smoothing using a standard deviation parameter (σ = 2.0) within the multi-dimensional image processing module of the SciPy library (version 1.13.0) [52]. The localized relative free energy, *G(X)*, of any specific state defined by the collective variable vector X was calculated utilizing the fundamental Boltzmann relationship:

$$G\left(X\right)=-k_{B}T\;lnP(X)$$

where *k_B_* is the Boltzmann constant and *T* is the temperature (310.15 K), and *P(X)* represents the normalized probability density of the system populating that specific coordinate state within the total sampled ensemble [53]. All subsequent topological rendering, free energy contour plotting, and thermodynamic basin visualizations were generated using the Matplotlib (version 3.10.0) and Seaborn (version 0.13.2) visualization libraries [54,55].

#### 3.5.2. Structural Validation and Ramachandran Plots

The overall structural integrity and backbone conformational stability of the hAChE enzyme complexed with lead compound were validated using dynamic Ramachandran analysis. This process monitored the time-dependent conservation of secondary structure elements under mutational stress. The underlying peptide backbone dihedral angles (ϕ and ψ) were extracted systematically for all non-terminal amino acid residues across every sampled frame of the production trajectories using specialized Python script architectures (Version 2.9.0) operating via the MD Analysis framework. Rather than relying on static, external validation servers, the individual residue coordinates were mapped and classified into Favored, Allowed, and Disallowed regions based directly on the canonical stereochemical boundary definitions encoded within the custom Python processing scripts. To move beyond traditional static validation paradigms, a temporal biophysical stress test was implemented in the form of a Residue Persistence Analysis applied to any coordinates fluctuating outside the core defined zones. This persistence profile tracked the fractional duration that a given amino acid occupied a disallowed dihedral state over the 100 ns simulation timeline. This mathematical tracking allowed the protocol to clearly distinguish between transient, entropically driven thermal fluctuations and stable, functionally relevant local structural strain, such as the rigid conformation required to maintain the active geometry of the catalytic residue Ser203.

### 3.6. Molecular Mechanics/Generalized Born Surface Area (MM/GBSA) Calculations

To obtain quantitative estimates of the absolute binding affinity and to isolate the specific driving forces governing mutational resilience, end-state Molecular Mechanics/Generalized Born Surface Area (MM/GBSA) calculations were performed. Given the extensive structural sampling generated across the independent triplicate trajectories for lead compound, a uniform temporal slicing protocol was executed using the MD Analysis library (version 2.7.0) [56]. Representative conformational frames were systematically extracted at a baseline interval of every 10 ns from the 100 ns production windows, providing a statistically balanced structural ensemble for implicit solvent calculations. The resulting protein–ligand snapshots were processed using the high-performance Uni-GBSA pipeline (version 2.4.2) [57]. The total binding free energy, Δ*G_total_*, was resolved utilizing the standard thermodynamic summation:

$${∆G}_{total}={∆E}_{vdw}+{∆E}_{elec}+{∆G}_{solv}-T∆S$$

where Δ*E_vdw_* and Δ*E_elec_* represent the gas-phase molecular mechanics van der Waals and electrostatic interaction energies. These were calculated utilizing the AMBER force field parameters (ff14SB for the protein matrix and GAFF2 for the ligand) assigned autonomously by the Uni-GBSA parameterization engine [58,59]. The total solvation free energy accounts for the combined polar and non-polar solvation contributions. The polar desolvation component was computed utilizing the advanced Onufriev–Bashford–Case (OBC_2_) Generalized Born implicit solvent model [60], parameterized with an operating flag of igb = 5. The electrostatic boundary conditions were established by setting the internal dielectric constant of the solute to 1.0 to reflect the hydrophobic core of the protein matrix, while the exterior bulk solvent dielectric constant was set to 80.0, adjusted to a physiological salt concentration of 0.150 M NaCl. To map the localized load shifting responsible for stabilizing the complexes upon the loss of major aromatic anchors, the total binding free energy was decomposed down to the individual amino acid level using the dedicated per-residue energy decomposition module within the Uni-GBSA framework, isolating the exact energetic contributions of neighboring stabilizers.

## 4. Conclusions

This study establishes a definitive, physics-based computational paradigm that shifts the boundary of neuroprotective drug discovery from static, single-residue dependencies to dynamic, multi-site target adaptation. By implementing an integrated generative-to-kinetic screening framework, we successfully looked beyond the conventional limitations of rigid molecular docking to discover small-molecule architectures capable of surviving targeted target-site mutations. Through explicit all-atom molecular dynamics, enhanced-sampling Metadynamics, and end-state binding free energy calculations, this investigation demonstrates that long-term target occupancy within the 20 Å human acetylcholinesterase (hAChE) gorge is governed by an intricate, interconnected relationship between kinetic stability, thermodynamic landscape depth, and mechanical allostery. The discovery of the alternative “Solvation Rescue” pose populated by Lead **1631** provides a compelling challenge to traditional biophysical models of drug resistance, proving that structural mutations can be energetically exploited rather than merely tolerated. Projecting the Gibbs free energy surfaces revealed that the W86A mutation actively forced the complex into a significantly deeper, lower-entropy energy funnel (ΔG_min_ = 9.23 ± 1.98 kJ/mol) than the native wild-type state (ΔG_min_ = 5.26 ± 1.69 kJ/mol), demonstrating that the ligand possesses the orientational plasticity required to substitute core aromatic interactions with expanded, water-mediated electrostatic networks.

Crucially, our Dynamic Cross-Correlation Matrix (DCCM) computations and free energy component decompositions mapped the exact mechanical rewiring networks that drive this thermodynamic adaptability. The structural deletion of primary side chains triggered a distinct mechanical inversion of the core enzyme framework, where the synchronized, in-phase movements across the wild-type gorge channel converted into highly coordinated, out-of-phase anti-correlated clamping fluctuations (−0.04) that structurally brace the ligand bridge between the peripheral entrance rim and the catalytic floor. This compensatory mechanism effectively rescues the binding affinity, ensuring that the net energetic penalty for losing a critical catalytic anchor is minimized to a negligible +1.59 kcal/mol along a perfect electrostatic-solvation see-saw diagonal. Collectively, these breakthroughs indicate that symmetrical scaffolds capable of allosterically rewiring enzyme vibrations represent a superior architectural strategy for engineering mutation-proof neurotherapeutics. The robust generative-to-kinetic pipeline validated in this work offers a highly predictive, scalable computational blueprint for prioritizing dynamically adaptable leads early in the development lifecycle, ensuring that future treatments for Alzheimer’s disease remain resilient against the heterogeneous landscapes and structural variations encountered in clinical environments. The proof and details supporting the figures presented in this manuscript are provided in the Supplementary Materials.

## Figures and Tables

**Table 1:** Predicted Binding Affinities and Mutational Penalties across hAChE Variants.

Ligand	WT Affinity (ΔG, kcal/mol)	W86A Affinity (ΔG, kcal/mol)	W86A Penalty (ΔΔG)	W286A Affinity (ΔG, kcal/mol)	W286A Penalty (ΔΔG)
**41**	−13.1	−12.8	0.3	−12.6	0.5
**1631**	−13	−11.9	1.1	−12	1
**1821**	−12.5	−11.2	1.3	−12.7	0
**3191**	−12.9	−12.4	0.5	−11.1	1.8

ΔG: Gibbs free energy of binding against PDB 7XN1/4EY7 coordinates; ΔΔG: Mutational penalty calculated as the difference between mutated and native wild-type affinities. WT: Native wild-type enzyme; W86A: CAS mutant; W286A: PAS mutant.

**Table 2:** Summary of Metadynamics Thermodynamic Barriers and Landscape Minima for Lead **1631**.

System	CV1 Global Min (Å)	Transition Barrier (kcal/mol)	Absolute Escape Barrier (kcal/mol)	Landscape Topography
Wild-Type	7.87	4.92	11.37	Singular Funnel
W86A	11.9	1.23	12.54	Broadened Metastable
W286A	13.32	1.93 *	9.69	Fractured Islands

CV1: Distance between the ligand protonated nitrogen and the alpha-carbon of residue 86 (Trp or Ala); Absolute Escape Barrier: The maximum free energy required for complete ligand dissociation into the bulk solvent matrix. * The W286A transition barrier represents the specific activation energy required to escape the mid-gorge buffer zone and enter the primary detachment state located at 22.01 Å.

**Table 3:** MM/GBSA Binding Free Energy Components for Ligand **1631** (kcal/mol).

System	ΔE_vdw_	ΔE_elec_	ΔG_solv_	ΔG_total_	ΔΔG_total_
Wild Type	−55.01 ± 3.86	−239.13 ± 26.15	249.87 ± 25.92	−44.27 ± 5.53	—
W86A	−52.30 ± 3.72	−269.23 ± 18.54	278.85 ± 18.03	−42.68 ± 5.81	+1.59
W286A	−53.00 ± 4.01	−237.77 ± 17.82	245.57 ± 17.33	−45.20 ± 6.21	−0.93

All values are reported in kcal/mol as the mean of triplicate trajectories ± standard deviation. ΔE_vdw_: van der Waals contribution; ΔE_elec_: electrostatic energy; ΔG_solv_: sum of polar and non-polar solvation energies; ΔG_total_: total binding free energy. ΔΔG_total_ represents the energetic penalty or gain relative to the wild-type system.

## Data Availability

The original data presented in the study are openly available in the GitHub repository at https://github.com/tusharpawar49/AChE_Mutation (accessed on 3 July 2026), which contains all source files, model configurations, and raw simulation outputs systematically organized across dedicated operational directories.

## Supplementary Materials

The following supporting information can be downloaded at https://www.mdpi.com/article/10.3390/ph19081169/s1. S1. QSAR Validation Set; S2. RMSD Data and Statistical Analysis; S3. RMSF Raw Data; S4. MMGBSA Raw Data for Ligand 1631; S5. FEL Analysis Data and Visualization for Ligand 1631; S6. Metadynamics Data for Ligand 1631; S7. Ramachandran Plot final Data for Ligand 1631.
