# Supplementary material for: QSAR-ML- and Metadynamics-Guided Design of Symmetrical Bis-Indanones to Overcome Mutational Anchor Loss in Acetylcholinesterase
Source: Pharmaceuticals (Basel). 2026 Jul 26;19(8):1169. doi: 10.3390/ph19081169 (PMC13516619; doi:10.3390/ph19081169)
Supplement: Supplementary file 1 [file pharmaceuticals-19-01169-s001.zip › pharmaceuticals-4396408-supplementary.pdf]

QSAR-ML and Metadynamics Guided Design of Symmetrical Bis-Indanones to Overcome Mutational Anchor Loss in Acetylcholinesterase

Ghazala Muteeb [1], Shrikant S. Nilewar [2] Mohammad Aatif [3], and Tushar Janardan Pawar [4,*]

[1] *Department of Nursing, College of Applied Medical Sciences, King Faisal University, Al-Ahsa 31982, Saudi Arabia; graza@kfu.edu.sa*

[2] *Department of Pharmaceutical Chemistry, Maliba Pharmacy College, Uka Tarsadia University, Bardoli 394350, Gujarat, India; shrinilewar@gmail.com*

[3] *Department of Public Health, College of Applied Medical Sciences, King Faisal University, Al-Ahsa, 31982, Saudi Arabia; maahmad@kfu.edu.sa*

[4] *Centro de Investigación, Universidad Anáhuac Querétaro, Circuito Universidades I, Fracción 2 S/N, Zibatá, El Marqués 76246, Querétaro, Mexico*

* *Correspondence: tushar.pawar@anahuac.mx*

S1. QSAR Validation Set

SMILES	Is Inhibitor	AD Distance	Inside AD
COc1cc2cc(C(=O)C3CCC[N+](C)(CC4CCCCO4)CC3)sc2cc1OC	TRUE	0.762887	TRUE
O=C(NCCC1CCN(Cc2ccccc2)CC1)c1cc(=O)c2ccccc2o1	TRUE	0.804348	TRUE
COCC[N+]1(C)CCCC(C(=O)c2cc3cc(OC)c(OC)cc3s2)CC1	TRUE	0.761364	TRUE
CC(CN1CCN(Cc2ccccc2)CC1)N(C(=O)c1cccc([N+](=O)[O-])c1)c1ccccn1	TRUE	0.823529	FALSE
COc1ccc2cc(C(=O)NCCCCCNc3c4c(nc5ccccc35)CCCC4)c(=O)oc2c1	TRUE	0.714286	TRUE
COc1cc2onc(CCC3CCN(Cc4ccccc4)CC3)c2cc1C	TRUE	0.735632	TRUE
Cc1ccc(S(=O)(=O)SCc2ccc(C(F)(F)F)cc2)cc1	TRUE	0.6	TRUE
O=C(NCCCCCCCNc1c2c(nc3ccccc13)CCCC2)c1cc2cc(OC(F)(F)F)ccc2oc1=O	TRUE	0.669811	TRUE
C[N+]1(Cc2ccccc2)CCC(CCC(=O)c2ccc3c(c2)OCCO3)CC1	TRUE	0.8	TRUE
COc1cc2oc(C(=O)OCCC3CCN(Cc4ccccc4)CC3)cc(=O)c2cc1OC	TRUE	0.725275	TRUE
COc1cc2c(cc1OC)C(=O)C(CC1(F)CCN(Cc3ccccc3F)CC1)C2	TRUE	0.724138	TRUE
COc1cc2c(cc1OC)C(=O)C(Cc1cccc(CN3CCCCC3)c1)C2	TRUE	0.792683	TRUE
Cc1ccc2oc(=O)c(C(=O)NCCCCCNc3c4c(nc5ccccc35)CCCC4)cc2c1	TRUE	0.740385	TRUE
NS(=O)(=O)c1ccc(C(=O)n2[nH]c(=O)c3cc([N+](=O)[O-])ccc3c2=O)cc1	TRUE	0.588235	TRUE
COc1ccc2c(c1)CCC(NS(=O)(=O)N(C)C)C2	TRUE	0.764706	TRUE
COc1cc2c(cc1OC)C(=O)C(=Cc1cccc(CN(C)C)c1)C2	TRUE	0.733333	TRUE
CN1C(=O)Cc2cc3onc(CCC4CCN(Cc5ccccc5)CC4)c3cc21	TRUE	0.714286	TRUE
COc1cc2nc(C(=O)NCCC3CCN(Cc4ccccc4)CC3)cc(O)c2cc1OC	TRUE	0.762376	TRUE
COc1cc2c(cc1OC)C(=O)C(CC1CCN(Cc3ccccc3)CC1)C2	TRUE	0.792683	TRUE
Cc1ccccc1NC(=O)Oc1ccc2c(c1)C1(C)CCOC1N2C	TRUE	0.704545	TRUE
O=C(Nc1cc(-c2cn(CCNc3c4c(nc5ccccc35)CCCC4)nn2)ccn1)Nc1cccc2c1C1CCCCN1C2=O	TRUE	0.744681	TRUE
COc1ccc2c(c1)CC(=CC1CCN(Cc3ccccc3)CC1)C2=O	TRUE	0.759036	TRUE
CC1=CC2Cc3nc4cc(Cl)ccc4c(NCCCCCCCNc4c5c(nc6ccccc46)CCCC5)c3C(C1)C2	TRUE	0.792793	TRUE
C#CCN(Cc1ccc2cccc(O)c2n1)C(C#N)CCC1CCN(Cc2ccccc2)CC1	TRUE	0.759259	TRUE
CCN(CCCCCC(=O)Nc1ccc(-c2ccc(NC(=O)CCCCCN(CC)Cc3ccccc3OC)cc2)cc1)Cc1ccccc1OC	TRUE	0.825	FALSE
CCNC(=O)Oc1ccc2c(c1)C1(C)CCN(C)C1N2C	TRUE	0.7375	TRUE
CCCCCCNc1c2c(nc3ccc(OC)cc13)CCCC2	TRUE	0.792683	TRUE
CCOC(O)=C1C(C)=Nc2nc3c(c(N)c2C1c1ccccc1)CCCC3	TRUE	0.709302	TRUE
CCOc1ccc(Cc2nc3cc(C(=O)NCCSSCCNc4c5c(nc6ccccc46)CCCC5)ccc3n2CCC(C)C)cc1	TRUE	0.769784	TRUE
COc1cc2oc(C(=O)NCCC3CCN(Cc4ccccc4)CC3)cc(=O)c2cc1OC	TRUE	0.765957	TRUE
CC(=O)c1ccccc1NC(=O)c1ccc(OCCCC[n+]2ccc3ccccc3c2)cc1	TRUE	0.788889	TRUE
CC1=CC2Cc3nc4cc(Cl)ccc4c(NCCCCCCNC(=O)C(N)Cc4c[nH]c5ccccc45)c3C(C1)C2	TRUE	0.804878	TRUE
COc1cc2c(cc1OC)C(=O)C(CC1(F)CCN(Cc3ccccc3)CC1)C2	TRUE	0.758621	TRUE
COc1cc2cc(C(=O)NCCCCCCCNc3c4c(nc5ccccc35)CCCC4)c(=O)oc2cc1OC	TRUE	0.721154	TRUE
COc1ccc2cc3[n+](cc2c1OCc1ccc(C(F)(F)F)cc1)CCc1cc2c(cc1-3)OCO2	TRUE	0.704082	TRUE
CC(C)(C)c1cc[n+](CCCCCCC[n+]2ccc(C(C)(C)C)cc2)cc1	TRUE	0.769231	TRUE
CN(C)S(=O)(=O)NC1CCc2ccc(O)cc2C1	TRUE	0.776119	TRUE
COc1ccc(C2CC(c3c4ccccc4cc4ccccc34)=NN2)cc1	TRUE	0.753623	TRUE

COc1cc2cc(C(=O)C3CCC[N+](C)(Cc4ccc([N+](=O)[O-])o4)CC3)sc2cc1OC	TRUE	0.666667	TRUE
O=C(NCCCCCCCNc1c2c(nc3ccccc13)CCCC2)c1cc2ccccc2oc1=O	TRUE	0.734043	TRUE
Oc1ccc(C2CC(c3c4ccccc4cc4ccccc34)=NN2)c(O)c1	TRUE	0.757143	TRUE
COc1ccc2oc(=O)c(C(=O)NCCCCCCNc3c4c(nc5ccccc35)CCCC4)cc2c1	TRUE	0.716981	TRUE
COc1cccc2c(CCC3CCN(Cc4ccccc4)CC3)noc12	TRUE	0.784091	TRUE
COc1cc2c(cc1OC)SC(C(=O)CCc1cc[n+](Cc3coc([N+](=O)[O-])c3)cc1)C2	TRUE	0.680851	TRUE
COc1cccc(CCCCCc2cccc(OC)[n+]2C)[n+]1C	TRUE	0.755556	TRUE
COc1ccccc1CN(C)CCCCCC(=O)N(C)CCCCCCCCN(C)C(=O)CCCCCN(C)Cc1ccccc1OC	TRUE	0.788732	TRUE
CCOC(O)=C1C(C)=Nc2nc3c(c(N)c2C1c1cccc(OC)c1)CCCC3	TRUE	0.732673	TRUE
c1cc[n+](Cc2ccc3ccc(C[n+]4ccccc4)cc3c2)cc1	TRUE	0.829268	FALSE
Cc1ccccc1NC(=O)Oc1ccc2c(c1)C1(C)CCN(C)C1N2C	TRUE	0.730337	TRUE
COc1cc(CNC(=O)CCCCCCNc2c3c(nc4cc(Cl)ccc24)CC2C=C(C)CC3C2)ccc1O	TRUE	0.781955	TRUE
Cc1ccccc1NC(=O)Oc1ccc2c(c1)C1(C)CCOC1O2	TRUE	0.714286	TRUE
CCOC(O)=C1C(C)=Nc2nc3c(c(N)c2C1c1cccc([N+](=O)[O-])c1)CCCC3	TRUE	0.666667	TRUE
CCC(C)n1cc(C(=O)C(F)F)cn1	TRUE	0.698113	TRUE
c1cc[n+](CCCCCCCCCCCC[n+]2cccc3ccccc32)cc1	TRUE	0.823529	FALSE
CN(CCC(=O)N1CCN(CCNc2c3c(nc4ccccc24)CCCC3)CC1)C1CCCCC1	TRUE	0.764151	TRUE
COc1cccc(CCCCCCCc2cccc(OC)[n+]2C)[n+]1C	TRUE	0.755556	TRUE
c1ccc2oc(CNCCCCCCCNc3c4c(nc5ccccc35)CCCC4)cc2c1	TRUE	0.795455	TRUE
CNC(=O)Oc1ccc2c(c1)C1(C)CCOC1N2C	TRUE	0.697368	TRUE
CN(CCCN1CCN(CCNc2c3c(nc4ccccc24)CCCC3)CC1)C1CCCCC1	TRUE	0.806122	TRUE
CCN(CC)Cc1cccc(CC2Cc3cc(OC)c(OC)cc3C2=O)c1	TRUE	0.782051	TRUE
COc1cc2cc(C(=O)C3CCC[N+](C)(Cc4ccccc4)CC3)sc2cc1OC	TRUE	0.758242	TRUE
NS(=O)(=O)c1ccc(C(=O)n2[nH]c(=O)c3c(F)cccc3c2=O)cc1	TRUE	0.59375	TRUE
O=C1Cc2cc3c(CCC4CCN(Cc5ccccc5)CC4)noc3cc2N1	TRUE	0.736264	TRUE
O=[N+]([O-])c1cccc(C2CC(c3c4ccccc4cc4ccccc34)=NN2)c1	TRUE	0.704225	TRUE
COC(=O)C[N+]1(C)CCC(CCC(=O)C2Cc3cc(OC)c(OC)cc3S2)CC1	TRUE	0.747126	TRUE
Cc1ccc2oc(-c3ccc(OCCCCCCN4CCCCC4)cc3)cc(=O)c2c1	TRUE	0.761364	TRUE
Cc1c(S(=O)(=O)c2ccc(Cl)cc2)csc1C(=O)NC1CCN(Cc2ccccc2)CC1	TRUE	0.670455	TRUE
COc1ccc2c(c1)CC(NC(=O)N(C)C)CC2	TRUE	0.768116	TRUE
COc1cc2c(cc1OC)C(=O)C(Oc1ccc(CN3CCN(C)CC3)cc1)C2	TRUE	0.756098	TRUE
CC(=O)Nc1ccc2c(CCC3CCN(Cc4ccccc4)CC3)noc2c1	TRUE	0.780488	TRUE
Cc1cc(C(=O)CCC2CC[N+](C)(Cc3ccccc3)CC2)nn1-c1ccccc1	TRUE	0.738095	TRUE
CC(=O)c1ccccc1NC(=O)c1ccc(OCCCCC[n+]2ccc3ccccc3c2)cc1	TRUE	0.791209	TRUE
Oc1ccc2c(CCC3CCN(Cc4ccccc4)CC3)noc2c1	TRUE	0.765432	TRUE
COc1cc2c(cc1OC)C(=O)C(=Cc1cccc(CN3CCCC3)c1)C2	TRUE	0.75	TRUE
CC(=O)c1ccccc1NC(=O)c1ccc(OCCCCCC[n+]2ccccc2)cc1	TRUE	0.765432	TRUE
NS(=O)(=O)c1ccc(C(=O)n2[nH]c(=O)c3c([N+](=O)[O-])cccc3c2=O)cc1	TRUE	0.633803	TRUE
c1ccc(C2CC(c3c4ccccc4cc4ccccc34)=NN2)cc1	TRUE	0.77193	TRUE
CN(C)S(=O)(=O)NC1CCc2c(O)cccc2C1	TRUE	0.764706	TRUE
O=C(NCCCCCCNc1c2c(nc3ccccc13)CCCC2)c1cc2cc(OC(F)(F)F)ccc2oc1=O	TRUE	0.669811	TRUE
CC(C)(C)c1cc[n+](C=C2C=C3CC(=C[n+]4ccc(C(C)(C)C)cc4)C=CC3=CC2)cc1	TRUE	0.811594	TRUE
COc1cc2cc(C(=O)C3CC[N+](C)(Cc4ccccc4)CCC3OC)sc2cc1OC	TRUE	0.752688	TRUE

FC(F)(F)Oc1ccc(CNC2CCN(Cc3ccccc3)C2)cc1	TRUE	0.753425	TRUE
CCc1ccccc1NC(=O)Oc1ccc2c(c1)C1(C)CC[N+](C)([O-])C1N2C	TRUE	0.760417	TRUE
COc1ccc(Cn2cc(C(=O)NCCCNc3c4c(nc5cc(Cl)ccc35)CCCC4)c(=O)c3ccccc32)cc1	TRUE	0.801653	TRUE
CCN(CC)c1cc(C=C2OC(=O)c3cc(OC)c(OC)cc32)ccc1O	TRUE	0.697368	TRUE
O=C(CNCc1cc(F)cc(F)c1)Nc1ccc2nc3n(c(=O)c2c1)CCC3	TRUE	0.682353	TRUE
CN(CCCN1CCN(CCNc2c3c(nc4ccccc24)CCCC3)CC1)c1ccccc1	TRUE	0.804124	TRUE
COP1(=O)OCC2COC(=O)C2=C(C)O1	TRUE	0.588235	TRUE
COc1cc2c(cc1OC)C[N+](C)(CCCCCCCc1cccc(OC)[n+]1C)CC2	TRUE	0.811765	TRUE
CCC(CC)n1cc(C(=O)C(F)F)cn1	TRUE	0.692308	TRUE
COc1cc(-c2cc3c(c(=O)o2)CC2(O)C(C)(CCC4(O)C(C)(C)C=CC(=O)C42C)O3)cc(OC)c1OC	TRUE	0.691489	TRUE
c1ccc(CN2CCC(CCc3noc4ccccc34)CC2)cc1	TRUE	0.753623	TRUE
COc1cccc(CCCCCCc2cccc(OC)[n+]2C)[n+]1C	TRUE	0.755556	TRUE
O=C(CCCCC1CCSS1)NCCCNc1c2c(nc3cc(Cl)ccc13)CCCC2	TRUE	0.766355	TRUE
CCCC(C)n1cc(C(=O)C(F)(F)F)cn1	TRUE	0.622642	TRUE
CCCC(C)n1cc(C(=O)C(F)F)cn1	TRUE	0.709091	TRUE
Nc1c2c(nc3c1C(c1cccc(Br)c1)NC(=S)N3)CCCC2	TRUE	0.7375	TRUE
COc1cc2c(cc1OC)C(=O)C(CC1(F)CCN(Cc3cc(F)c(F)cc3F)CC1)C2	TRUE	0.717647	TRUE
O=C(CN1CCN(Cc2ccccc2)CC1)Nc1ccc(C(F)(F)F)cc1Cl	TRUE	0.69863	TRUE
NS(=O)(=O)c1ccc(C(=O)n2[nH]c(=O)c3ccc(C(=O)O)cc3c2=O)cc1	TRUE	0.651515	TRUE
CC(=O)Oc1ccccc1C(=O)O	FALSE	0.851064	FALSE
CC(C)Cc1ccc(cc1)C(C)C(=O)O	FALSE	0.851852	FALSE
CC(=O)Nc1ccc(O)cc1	FALSE	0.846154	FALSE
COc1ccc2cc(ccc2c1)C(C)C(=O)O	FALSE	0.851852	FALSE
O=C(O)Cc1ccccc1Nc2c(Cl)cccc2Cl	FALSE	0.842857	FALSE
CC(C(=O)O)c1cccc(C(=O)c2ccccc2)c1	FALSE	0.859649	FALSE
Cc1ccc(cc1)c2cc(nn2c3ccc(cc3)S(=O)(=O)N)C(F)(F)F	FALSE	0.815385	TRUE
COc1ccc2c(c1)c(CC(=O)O)c(C)n2C(=O)c3ccc(Cl)cc3	FALSE	0.849315	FALSE
CCOc1ccc(NC(C)=O)cc1	FALSE	0.838235	FALSE
O=C(O)c1ccccc1O	FALSE	0.883721	FALSE
[H]O[H]	FALSE	0.981481	FALSE
CCO	FALSE	0.925	FALSE
CO	FALSE	0.92	FALSE
CC(C)O	FALSE	0.925	FALSE
CC(=O)C	FALSE	0.875	FALSE
CCOC(C)=O	FALSE	0.87037	FALSE
CS(C)=O	FALSE	0.928571	FALSE
Cc1ccccc1	FALSE	0.891892	FALSE
CCCCCC	FALSE	0.9375	FALSE
ClC(Cl)Cl	FALSE	0.962963	FALSE
NCC(=O)O	FALSE	0.891304	FALSE
CC(N)C(=O)O	FALSE	0.886364	FALSE
CC(C)C(N)C(=O)O	FALSE	0.877551	FALSE
CC(C)CC(N)C(=O)O	FALSE	0.877551	FALSE

CCC(C)C(N)C(=O)O	FALSE	0.865385	FALSE
OCC(N)C(=O)O	FALSE	0.87234	FALSE
CC(O)C(N)C(=O)O	FALSE	0.87234	FALSE
SCC(N)C(=O)O	FALSE	0.895833	FALSE
CSCCC(N)C(=O)O	FALSE	0.884615	FALSE
O=C(O)C1CCCN1	FALSE	0.901961	FALSE
NC(Cc1ccccc1)C(=O)O	FALSE	0.857143	FALSE
NC(Cc1ccc(O)cc1)C(=O)O	FALSE	0.862069	FALSE
NC(Cc1c[nH]c2ccccc12)C(=O)O	FALSE	0.858974	FALSE
NC(CC(=O)O)C(=O)O	FALSE	0.882353	FALSE
NC(CCC(=O)O)C(=O)O	FALSE	0.886792	FALSE
NC(CC(N)=O)C(=O)O	FALSE	0.884615	FALSE
NC(CCC(N)=O)C(=O)O	FALSE	0.882353	FALSE
NC(Cc1c[nH]cn1)C(=O)O	FALSE	0.859649	FALSE
NCCCCC(N)C(=O)O	FALSE	0.890909	FALSE
NC(N)=NCCCC(N)C(=O)O	FALSE	0.87931	FALSE
C(C1C(C(C(C(O1)O)O)O)O)O	FALSE	0.921875	FALSE
C(C1C(C(C(O1)(CO)O)O)O)O	FALSE	0.898551	FALSE
C1C(C(C(C(O1)O)O)O)O	FALSE	0.904762	FALSE
C1C(C(C(C(O1)O)O)O)O	FALSE	0.904762	FALSE
C(C1C(C(C(C(O1)O)O)O)O)O	FALSE	0.921875	FALSE
OCC(O)CO	FALSE	0.930233	FALSE
OCC(O)C(O)C(O)C(O)CO	FALSE	0.933333	FALSE
OCC(O)C(O)C(O)CO	FALSE	0.933333	FALSE
OCC(O)C(O)CO	FALSE	0.930233	FALSE
OCC(O)C(O)C(O)C(O)CO	FALSE	0.933333	FALSE
OCC(O)C1OC(=O)C(O)=C1O	FALSE	0.886076	FALSE
O=C(O)c1cccnc1	FALSE	0.844444	FALSE
NC(=O)c1cccnc1	FALSE	0.847826	FALSE
CC(C)(CO)C(O)C(=O)NCCC(=O)O	FALSE	0.862069	FALSE
Cc1ncc(CO)c(CO)c1O	FALSE	0.87931	FALSE
C1C2C(C(S1)CCCCC(=O)O)NC(=O)N2	FALSE	0.883117	FALSE
CC1=CC2=C(C=C1C)N(CC(O)C(O)C(O)CO)C3=NC(=O)NC(=O)C3=N2	FALSE	0.863014	FALSE
CC1=CC(=O)c2ccccc2C1=O	FALSE	0.869565	FALSE
CC1=C(C(CCC1)(C)C)C=CC(=CC=CC(=CCO)C)C	FALSE	0.883721	FALSE
NC(Cc1cc(I)c(Oc2cc(I)c(O)c(I)c2)c(I)c1)C(=O)O	FALSE	0.867647	FALSE
OC(=O)CC(O)(C(=O)O)CC(=O)O	FALSE	0.87234	FALSE
CC(O)C(=O)O	FALSE	0.883721	FALSE
OC(CC(=O)O)C(=O)O	FALSE	0.893617	FALSE
OC(C(O)C(=O)O)C(=O)O	FALSE	0.906977	FALSE
O=C(O)c1ccccc1	FALSE	0.842105	FALSE
CC=CC=CC(=O)O	FALSE	0.888889	FALSE
CCC(=O)O	FALSE	0.880952	FALSE

O=C1NS(=O)(=O)c2ccccc12	FALSE	0.893939	FALSE
COc1cc(C=O)ccc1O	FALSE	0.838235	FALSE
O=C([O-])CCC(N)C(=O)O.[Na+]	FALSE	0.892857	FALSE
Cc1cc(NS(=O)(=O)c2ccc(N)cc2)no1	FALSE	0.885714	FALSE
COc1cc(Cc2cnc(N)nc2N)cc(OC)c1OC	FALSE	0.861111	FALSE
NNC(=O)c1ccncc1	FALSE	0.84	FALSE
NC(=O)c1cnccn1	FALSE	0.869565	FALSE
C1=CC(=CC=C1C(C(CO)NC(=O)C(Cl)Cl)O)[N+](=O)[O-]	FALSE	0.84	FALSE
CC(=O)NCC1CN(C(=O)O1)c2ccc(F)c(N3CCOCC3)c2	FALSE	0.84375	FALSE
O=C(O)c1cn(C2CC2)c2cc(N3CCNCC3)c(F)cc2c1=O	FALSE	0.851852	FALSE
CCN1C=C(C(=O)c2cnc(C)cc21)C(=O)O	FALSE	0.84375	FALSE
Cc1ncc([N+](=O)[O-])n1CCO	FALSE	0.8375	FALSE
OC(Cn1cncn1)(Cn2cncn2)c3ccc(F)cc3F	FALSE	0.822581	TRUE
CN(C)C(=N)NC(=N)N	FALSE	0.910714	FALSE
CCOC(=O)C1=C(COCCN)NC(C)=C(C(=O)OC)C1c2ccccc2Cl	FALSE	0.835165	FALSE
CCCCC(=O)N(Cc1ccc(cc1)c2ccccc2c3nnn[nH]3)C(C(C)C)C(=O)O	FALSE	0.869048	FALSE
CC(C)NCC(O)COc1ccc(CC(N)=O)cc1	FALSE	0.855422	FALSE
CC(C)NCC(O)COc1cccc2ccccc12	FALSE	0.882353	FALSE
CC(CS)C(=O)N1CCCC1C(=O)O	FALSE	0.873016	FALSE
NS(=O)(=O)c1cc2c(cc1Cl)NCNS2(=O)=O	FALSE	0.861111	FALSE
CN1C=NC2=C1C(=O)N(C(=O)N2C)C	FALSE	0.839286	FALSE
NCCC1(CC(=O)O)CCCCC1	FALSE	0.903614	FALSE
COc1ccc2[nH]c(S(=O)Cc3ncc(C)c(OC)c3C)nc2c1	FALSE	0.870968	FALSE
CC1CCC(C(C1)O)C(C)C	FALSE	0.90566	FALSE
CC1(C)C2CCC1(C)C(=O)C2	FALSE	0.890909	FALSE
COc1cc(CC=C)ccc1O	FALSE	0.845238	FALSE
O=CC=Cc1ccccc1	FALSE	0.907407	FALSE
CC1=CCC(C(=C)C)CC1	FALSE	0.887324	FALSE
CC(=CCCC(C)(C=C)O)C	FALSE	0.909091	FALSE
Cc1ccc(C(C)C)c(O)c1	FALSE	0.891304	FALSE
c1ccc2ccccc2c1	FALSE	0.9	FALSE
c1ccc2cc3ccccc3cc2c1	FALSE	0.90625	FALSE
Cc1cc(C(C)(C)C)c(O)c(C(C)(C)C)c1	FALSE	0.857143	FALSE

(C) Lead Mapping: Lead 1631 Structural Reliability

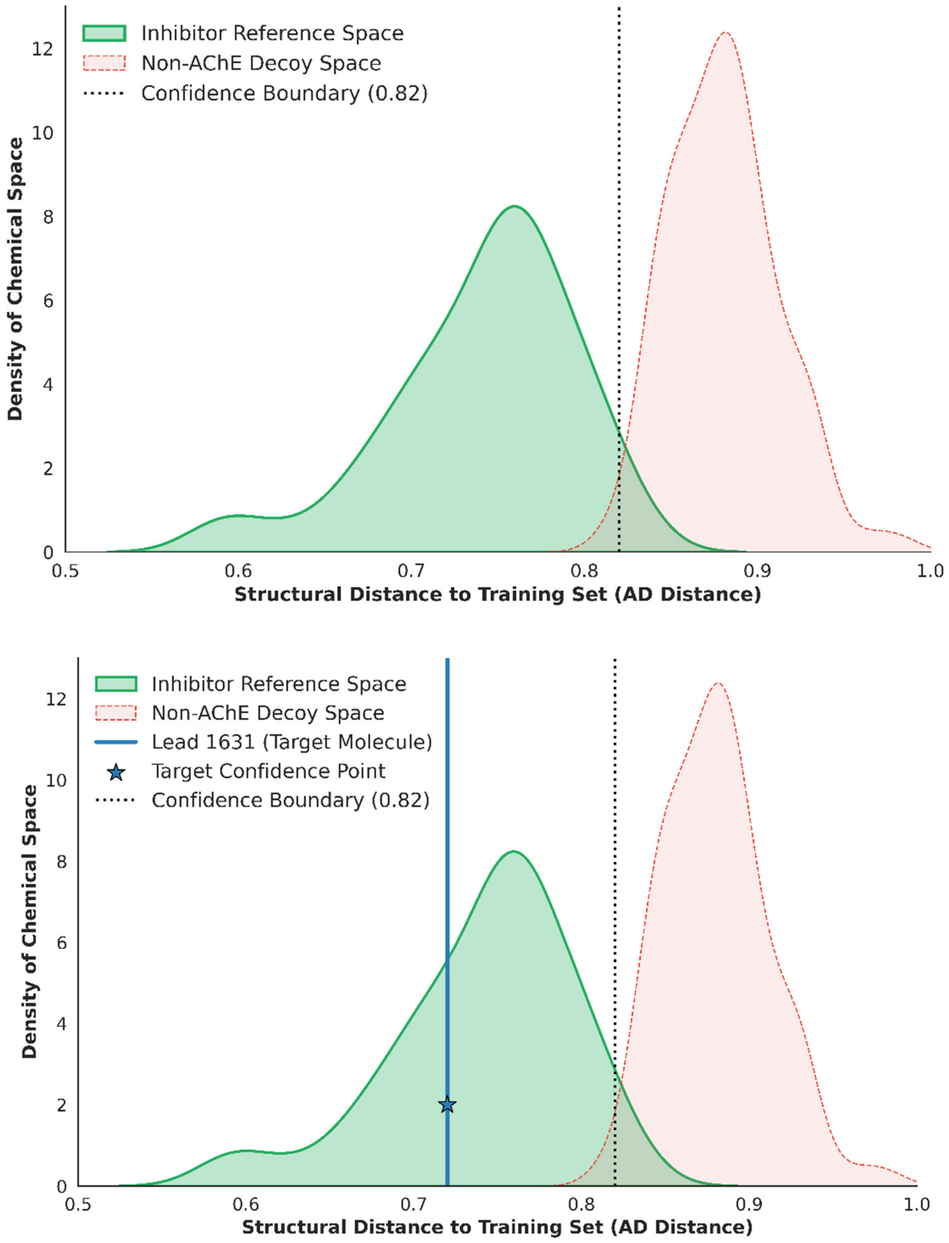

S2. RMSD Data and Statistical Analysis

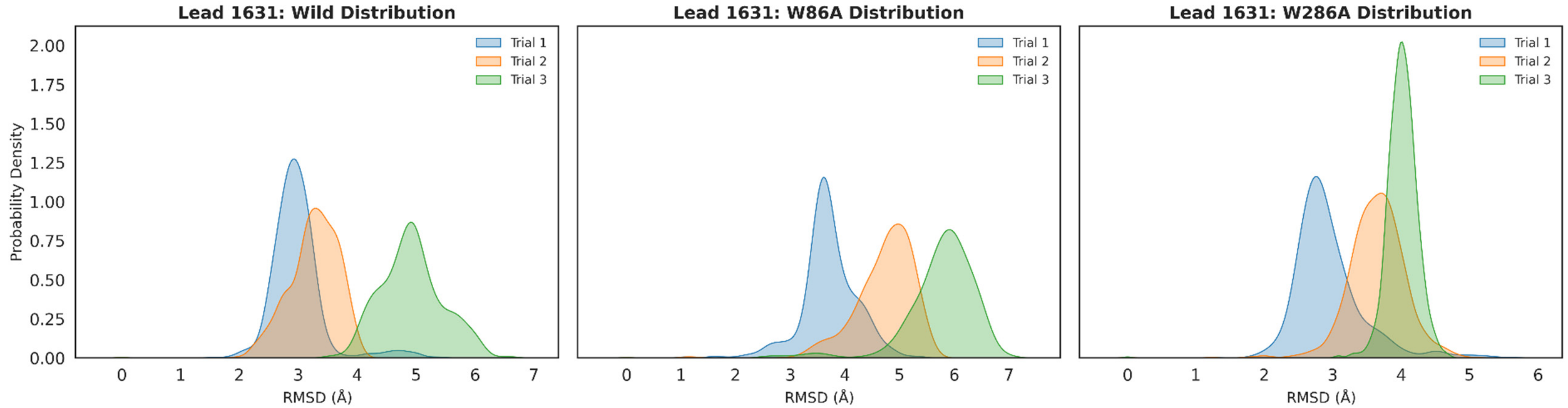

	Wild **41**	Wild **1821**	Wild **3191**	W86A **41**	W86A **1821**	W86A **3191**	W286A **41**	W286A **1821**	W286A **3191**
AVG	2.668933	2.888996	4.289634	8.115609	6.534997	5.384246	4.580683	5.254235	2.858987
SD	0.271777	0.677541	0.466157	1.651661	0.88559	1.88715	0.908903	0.573864	0.536385
Slope-50	-0.00913	0.009134	-0.00528	0.042	-0.01539	0.009198	0.003154	-0.00611	0.018643
R2	0.220746	0.324781	0.100794	0.350671	0.211244	0.015016	0.016183	0.079089	0.472546
CV	10.18297	23.45248	10.86705	20.35166	13.5515	35.04948	19.84208	10.92193	18.76136
Correlation	0.30538	0.872828	0.52339	0.811812	0.695616	0.893416	0.744786	0.354201	0.564757
Frequency	6	1	1	1	1	1	1	1	1

	**Wild**	**Wild**	**Wild**	**Wild**	**Wild**

	1	2	3	AVG	SEM
AVG	2.982774	3.267439	4.928728	3.726314	0.644098
SD	0.45316	0.404342	0.520844	0.265831	0.156998
Slope-50	0.002777	-0.0024	-0.00073	-0.00012	
R2	0.038335	0.007448	0.000557	5.18E-05	
CV	15.19258	12.3749	10.5675	7.133877	
Correlation	0.232534	0.456774	0.690429	0.502319	
Frequency	1	6	3	1	

	W86A 1	W86A 2	W86A 3	W86A AVG	W86A SEM	W286A 1	W286A 2	W286A 3	W286A AVG	W286A SEM
AVG	3.711607	4.728656	5.778129	4.739464	0.62717	2.93915	3.650601	4.017405	3.535719	0.370801
SD	0.501155	0.534695	0.629397	0.429707	0.160419	0.496646	0.400574	0.204479	0.206298	0.127267
Slope-50	-0.00726	-0.01389	-0.00277	-0.00797		-0.0007	-0.00952	0.0054	-0.0016	
R2	0.163794	0.470343	0.007397	0.289303		0.000393	0.152326	0.227372	0.014528	
CV	13.50236	11.30755	10.89275	9.066571		16.8976	10.97283	5.089819	5.834686	
Correlation	0.529432	0.686529	0.237564	0.678664		0.122503	0.285574	0.100422	0.025473	
Frequency	1	1	1	1		1	1	1	1	

S3. RMSF Raw Data

Atom#	Wild 41	W86A 41	W286A 41	Wild 1821	W86A 1821	W286A 1821	Wild 3191	W86A 3191	W286A 3191
1	0.698	2.491	1.031	0.944	1.243	1.251	0.724	2.138	1.374
2	0.621	2.179	0.88	0.955	1.359	1.604	0.793	2.201	1.494
3	0.584	1.951	0.886	0.931	1.379	1.529	0.69	2.268	1.402
4	0.526	1.877	0.942	0.901	1.184	1.293	1.17	3.483	2.081
5	0.546	1.781	0.859	0.948	1.487	1.528	1.01	2.991	1.729
6	0.615	2.037	0.972	0.942	1.422	1.58	0.907	2.8	1.629
7	0.652	2.29	0.921	1.036	1.277	1.376	0.821	2.454	1.426
8	0.55	1.795	0.823	1.362	1.338	1.331	0.757	2.331	1.375
9	0.526	1.912	1.034	1.452	1.636	1.12	0.747	2.146	1.37
10	0.525	2.236	1.25	1.31	1.488	0.99	0.835	2.249	1.579
11	0.57	2.709	1.656	1.322	1.32	1.075	0.895	2.417	1.643
12	0.663	3.181	2.011	1.161	1.285	0.939	0.877	2.451	1.495
13	0.619	2.943	1.893	1.166	1.214	1.011	0.98	2.714	1.617
14	0.641	2.707	1.82	1.107	1.317	1.017	1.033	2.917	1.663
15	0.788	3.231	2.303	1.212	1.374	1.181	1.105	3.009	1.899
16	0.599	2.161	1.41	1.234	1.565	1.251	1.318	3.276	2.087
17	0.821	2.431	1.118	1.104	1.632	1.117	1.528	3.759	2.49
18	0.71	2.783	1.092	1.03	1.483	0.951	0.672	2.413	1.444
19	2.099	4.089	3.392	1.01	1.551	0.838	0.707	2.399	1.345
20	1.783	3.615	2.862	1.084	1.44	0.828	0.74	2.116	1.562
21	1.582	3.359	2.568	1.182	1.545	0.852	0.695	2.106	1.542
22	1.264	2.934	1.981	0.996	1.316	1.307	0.672	2.315	1.844
23	1.025	2.701	1.543	1.131	1.225	1.184	0.649	2.733	1.559
24	0.825	2.681	1.364	1.208	1.449	1.593	0.804	2.168	1.585
25	0.679	2.767	1.273	1.446	1.5	1.565	0.861	2.597	1.402
26	0.735	3.125	1.778	1.551	1.406	1.051	1.098	3.34	1.506
27	0.893	3.218	2.038	1.355	1.281	1.067	1.277	4.032	1.547
28	0.936	2.931	1.773	1.907	1.706	0.939	1.208	4.086	1.461
29	1.193	3.096	2.092	2.776	2.069	0.922	0.993	3.567	1.356
30	1.326	3.066	2.138	3.158	2.527	0.951	0.836	2.817	1.347
31	1.642	3.436	2.617	2.774	2.598	1.019			
32				3.228	3.093	1.172			
33				2.427	2.262	1.1			
34				2.033	1.844	1.047			
35				2.464	1.766	1.261			

	Wild			W86A			W286A		
**Atom#**	**1**	**2**	**3**	**1**	**2**	**3**	**1**	**2**	**3**
1	1.307	1.562	1.756	2.292	1.374	1.215	1.053	0.787	0.621
2	1.184	1.407	1.681	2.2	1.023	1.083	1.029	0.784	0.621
3	1.051	1.232	1.513	1.718	0.832	1.028	1.012	0.797	0.583
4	1.157	1.289	1.53	1.894	1.023	1.121	1.04	0.826	0.616
5	1.301	1.495	1.67	2.353	1.354	1.285	1.067	0.85	0.656
6	1.326	1.595	1.724	2.294	1.519	1.324	1.066	0.828	0.627
7	1.302	1.224	1.585	2.13	1.057	1.103	1.156	0.961	0.699
8	1.156	1.035	1.508	1.595	0.937	0.97	1.156	1.005	0.637
9	1.023	1.087	1.502	1.647	0.738	0.953	1.077	0.917	0.602
10	1.096	1.147	1.668	2.185	0.809	0.968	1.158	1.046	0.685
11	2.183	2.546	2.692	3.806	2.236	1.682	1.391	1.133	1.377
12	1.826	2.207	2.325	3.107	1.892	1.506	1.193	0.958	1.186
13	1.59	1.787	2.036	2.951	1.702	1.311	1.188	0.889	0.71
14	1.974	1.858	2.378	3.574	1.944	1.372	1.923	1.035	1.383
15	1.539	1.824	1.915	2.777	1.927	1.526	1.179	0.976	0.703
16	1.125	0.862	1.471	1.22	1.097	0.994	1.242	1.117	0.686
17	1.089	0.754	1.206	1.18	1.258	0.923	1.062	1.073	0.694
18	1.006	0.693	1.229	1.406	1.267	0.875	1.106	1.219	0.645
19	1.111	0.698	1.194	1.622	1.195	0.777	1.102	1.578	0.666
20	1.097	0.644	1.018	1.368	1.589	0.713	1.149	1.237	0.735
21	1.011	0.58	0.942	1.651	1.723	0.736	1.235	1.012	0.705
22	0.961	0.638	1.006	2.154	2.073	0.857	1.388	1.426	0.72
23	0.971	0.675	1.232	2.158	1.738	0.914	1.308	1.317	0.697
24	1.07	0.654	0.929	1.853	1.521	0.855	1.541	1.319	0.882
25	0.948	0.641	0.9	1.379	1.35	0.998	1.866	1.15	1.213
26	1.16	0.61	0.907	1.539	1.344	1.31	2.22	1.392	1.919
27	1.265	0.669	0.971	1.757	1.483	1.76	2.782	1.626	2.824
28	1.215	0.788	1.047	1.754	1.772	1.967	3.046	1.698	3.209
29	1.103	0.895	1.124	1.795	2.11	1.828	2.85	1.816	2.972
30	0.952	0.821	1.048	1.666	1.88	1.368	2.271	1.568	2.077

S4. MMGBSA Raw Data for Ligand 1631

System	Residue	Mean Energy (kcal/mol)	Std Dev
W286A	ASP74	1.452012	1.074045
W286A	GLU202	-2.49033	2.29851
W286A	PHE338	-0.94542	0.338467
W286A	SER125	-0.96356	0.338067
W286A	SER203	-0.77964	0.177531
W286A	TRP286	-0.99325	0.32427
W286A	TRP86	-2.92757	1.184528
W286A	TYR124	-1.63013	0.459879
W286A	TYR337	-0.91546	0.778196
W286A	TYR341	-1.8904	0.867683
W286A	TYR72	-1.41013	0.478688
W86A	ASP74	-0.91607	0.989201
W86A	GLU202	0.95066	0.36496
W86A	PHE295	-1.97135	0.249688
W86A	PHE338	-0.90486	0.165157
W86A	SER125	0.86441	1.02826
W86A	TRP286	-2.53101	1.110312
W86A	TRP86	-2.45157	1.344023
W86A	TYR124	-1.65489	0.839357
W86A	TYR133	0.047629	0.77348
W86A	TYR337	-0.77317	0.414163
W86A	TYR341	-1.88514	0.934891
W86A	TYR72	-2.40816	0.917977
Wild Type	ASP74	1.560127	0.895492
Wild Type	PHE338	-2.09773	1.277274
Wild Type	SER125	-0.45808	1.762331
Wild Type	TRP286	-1.57801	0.564957
Wild Type	TRP86	-1.29332	0.454133
Wild Type	TYR124	-0.58948	0.912293
Wild Type	TYR337	-0.74378	0.174333
Wild Type	TYR341	-2.77549	0.924865
Wild Type	TYR72	-1.37465	0.681553

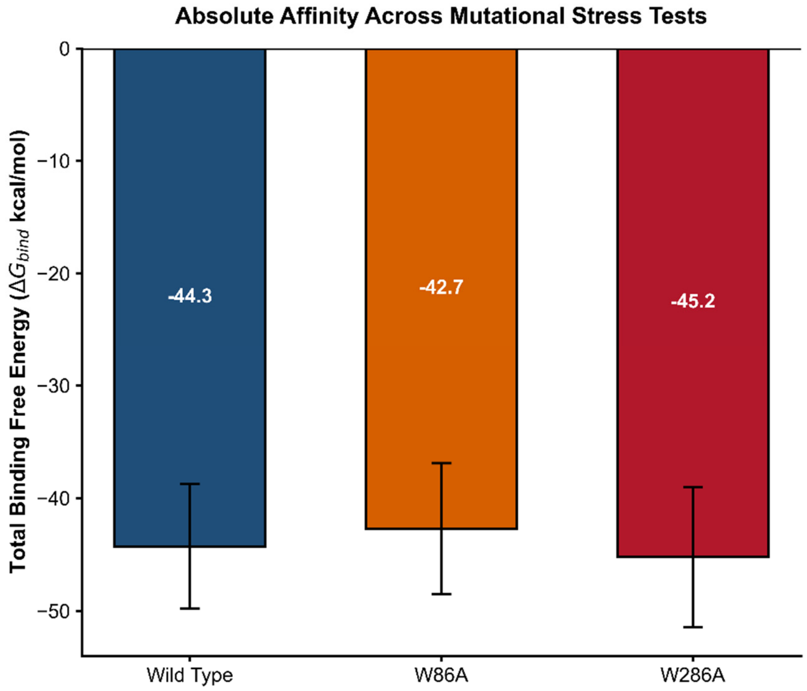
Absolute Affinity Across Mutational Stress Tests
Total Binding Free Energy (ΔG_bind kcal/mol)
0
−10
−20
−30
−40
−50
-44.3
-42.7
-45.2
Wild Type
W86A
W286A

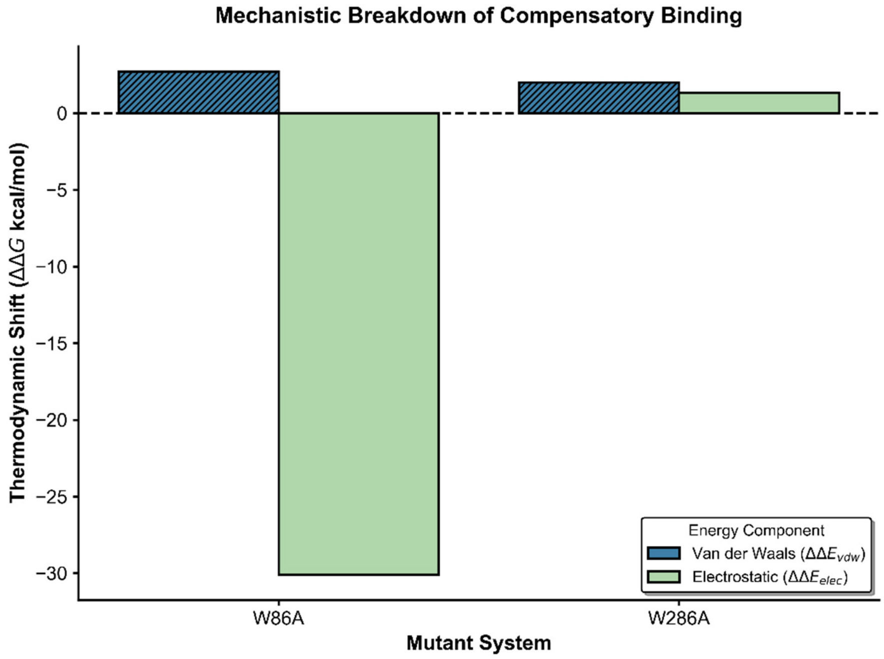
Mechanistic Breakdown of Compensatory Binding
Thermodynamic Shift (ΔΔG kcal/mol)
0
−5
−10
−15
−20
−25
−30
Energy Component
Van der Waals (ΔΔE_vdw)
Electrostatic (ΔΔE_elec)
W86A
W286A
Mutant System

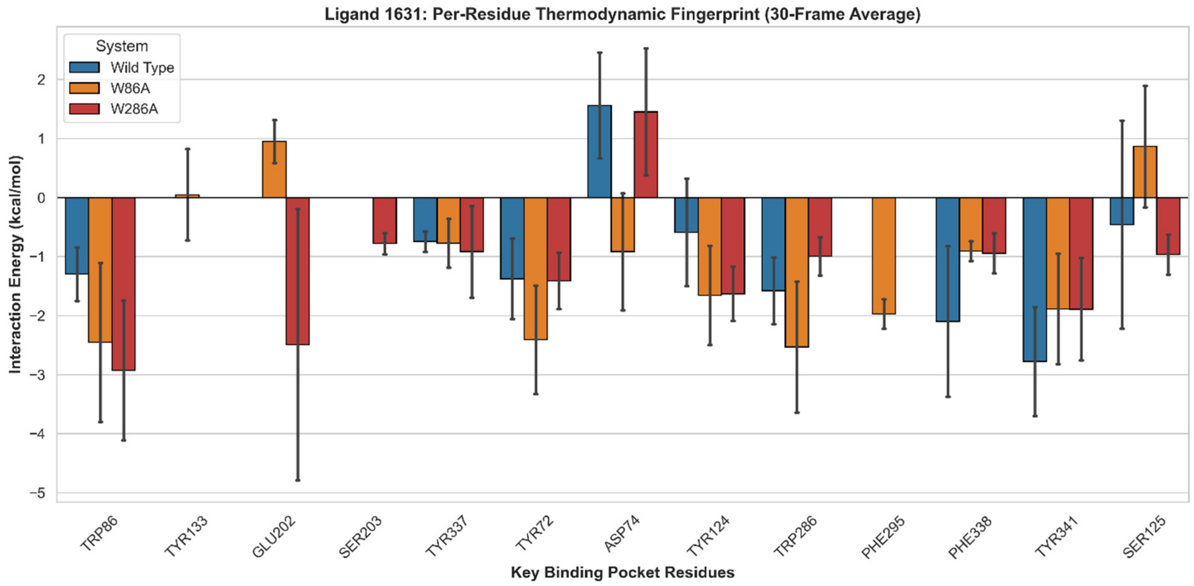
Ligand 1631: Per-Residue Thermodynamic Fingerprint (30-Frame Average)
System
Wild Type
W86A
W286A
Interaction Energy (kcal/mol)
2
1
0
−1
−2
−3
−4
−5
TRP86
TYR133
GLU202
SER203
TYR337
TYR72
ASP74
TYR124
TRP286
PHE295
PHE338
TYR341
SER125
Key Binding Pocket Residues

S5. FEL Analysis Data and Visualization for Ligand 1631

System	Mutation	Ligand ID	Replica	Global_Min RMSD Å	Global_Min Rg Å	ΔG min kJmol
Wild	None	**1631**	1	1.286	22.89	6.94
Wild	None	**1631**	2	1.475	23.01	3.557
Wild	None	**1631**	3	1.442	22.992	5.282
Mutated	W286A	**1631**	1	1.183	22.963	10.199
Mutated	W286A	**1631**	2	1.393	23.055	5.714
Mutated	W286A	**1631**	3	1.395	23.057	4.003
Mutated	W86A	**1631**	1	1.395	22.977	11.12
Mutated	W86A	**1631**	2	1.675	23.014	9.39
Mutated	W86A	**1631**	3	1.235	23.052	7.171

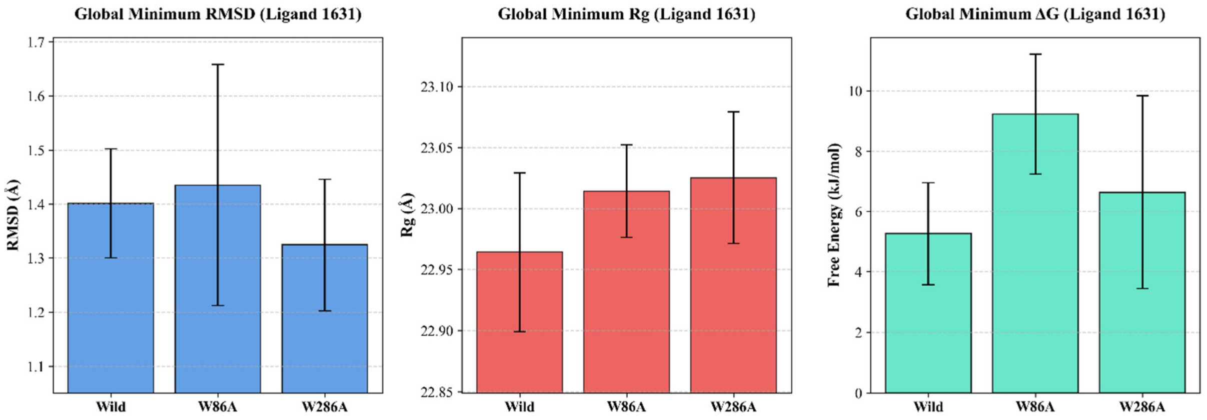

S6. Metadynamics Data for Ligand 1631

System	1D CAS Profile (CV1)	1D PAS Profile (CV2)	Kinetic Trajectory (150 ns)	Biophysical Inference (Thermodynamics)
**Wild-Type AChE**	**Global Min:** 7.87 Å; Transition Barrier: 4.92 kcal/mol (at 10.24 Å); Escape Barrier: 11.37 kcal/mol	**Global Min:** 11.02 Å; Transition Barrier: 4.96 kcal/mol (at 8.75 Å); Escape Barrier: >10.65 kcal/mol	**Stable Breathing:** Ligand demonstrates dynamic flexibility, smoothly exploring secondary compressed states. Convergence: Rapid rebinding following a 120 ns excursion.	**Robust Dual-Site Affinity:** 1D profiles mathematically confirm synchronized energy wells. High internal transition barriers (~4.9 kcal/mol) prove the ligand is tightly held in its primary pose, requiring significant energy to shift into metastable resting states before facing the massive absolute escape barriers.
**W86A Mutant (CAS Stress Test)**	**Global Min:** Shifts outward to 11.90 Å; Transition Barrier: Drops to 1.23 kcal/mol; Escape Barrier: 12.54 kcal/mol	**Global Min:** Shifts outward to 16.52 Å; Transition Barrier: Drops to 0.88 kcal/mol; Escape Barrier: 12.51 kcal/mol	**High Internal Volatility:** Ligand undergoes rapid, wide-ranging coordinate sampling. Confinement: CV bounds remain heavily restricted from complete solvent escape.	**Loss of Lock & Compensatory Tethering:** Deleting the Trp86 interaction abolishes the internal speed bumps. Transition barriers vanish (<1.3 kcal/mol), proving the ligand slides loosely and freely within the lower gorge. However, the system exhibits compensatory tethering; the intact PAS anchor completely traps the tumbling ligand, resulting in a massive absolute escape barrier (~12.5 kcal/mol) despite the lack of a specific CAS lock.
**W286A Mutant (PAS Stress Test)**	**Global Min:** Shifts outward to 13.32 Å; Secondary Min: Detached at 22.01 Å (ΔG = 1.93); Escape Barrier: Drops to 9.69 kcal/mol	**Global Min:** 12.29 Å; Compression Mins: Deeply shifted to 5.69 Å and 8.18 Å; Escape Barrier: Drops to 9.79 kcal/mol	**Gorge Detachment:** CV1 heavily explores extreme solvent extensions (>22 Å); Deep Compression: CV2 crashes deeply into the gorge interior (<6 Å).	**Breakdown of Energetic Cooperativity:** Removing Trp286 allows the indanone core to crash deeply into the gorge, structurally contorting the scaffold. Without the PAS anchor, the CAS anchor fails entirely. The NH+ moiety slides out of the binding site (CV1 > 22 Å), and the absolute escape barrier collapses below 10 kcal/mol, proving dual-site engagement is strictly cooperative.

System (Ligand Source)	Precise Visual Findings (from FES Contours)	Biophysical Inferences for Ligand 1631
**Wild-Type AChE**	**Deepest Basin:** Defined strictly by the **1.4 kcal/mol** contour. Primary Coordinates: Strongly localized at CV1 = 7.0–9.0 Å and CV2 = 10.5–14.5 Å. Secondary State: A broad, shallower basin (4.3–5.7 kcal/mol) exists at extended CV1 (10.0–17.5 Å) and compressed CV2 (5.0–8.0 Å). **Max Barrier: ~11.0 kcal/mol.**	**Primary Dual-Site Lock:** The ligand establishes a highly localized, stable dual-site interaction. The NH+ anchors tightly at the CAS (~8 Å), while the indanone interacts with the PAS (~12.5 Å).
**W86A Mutant (CAS Stress Test)**	**Deepest Basin:** Single, highly stable state defined by the **1.6 kcal/mol** contour. Primary Coordinates: CV1 = 8.0–13.5 Å, CV2 = 13.0–18.0 Å. Secondary State: A distinct local minimum appears at 4.7 kcal/mol, compressed to CV1 = 6.5–10.0 Å and CV2 = 9.0–12.0 Å. Max Barrier: Highest of all systems at ~12.5 kcal/mol.	**Broadened Metastable Basin:** Stripping the CAS anchor (Trp86) does not shatter the binding pose, but it removes the tight orientational lock. The binding territory expands significantly, indicating the ligand shifts into a broadened, looser conformation while remaining trapped deep inside the gorge.
**W286A Mutant (PAS Stress Test)**	**Deepest Basins:** Fractured into three distinct **1.2 kcal/mol** islands located across CV1 (8.0–14.5 Å) and CV2 (10.0–16.0 Å). Secondary States: Three new 2.4 kcal/mol basins appear, notably at extreme CV1 extensions (up to 22.5 Å) and extreme CV2 compressions (down to 5.5 Å). Max Barrier: Drops significantly to ~9.0 kcal/mol.	**Global Destabilization & Landscape Fracture:** Removing the Trp286 PAS anchor shatters the landscape. The indanone core tumbles through multiple discrete, sub-optimal poses, causing the entire scaffold to physically stretch and migrate toward the gorge entrance.

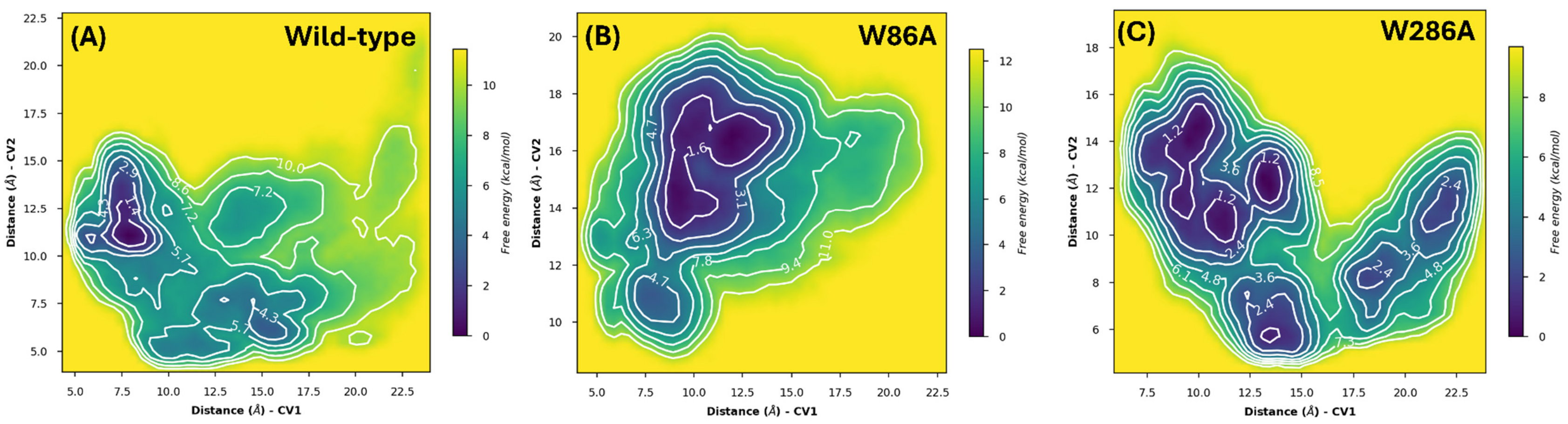

(A)
Wild-type
(B)
W86A
(C)
W286A
Distance (Å) - CV1
Distance (Å) - CV2
Free energy (kcal/mol)

S7. Ramachandran Plot final Data for Ligand 1631

System	Mutation	Ligand ID	Replica	Favoured (%)	Allowed (%)	Disallowed (%)	Disallowed Residues (>5% time)
Mutated	W286A	1631	1	82.88	15.38	1.74	GLY14, GLY15, GLY26, GLY27, GLY43, GLY58, GLY121, GLY126, GLY154, GLY201, SER203, GLY230, GLY234, GLY260, GLY261, GLY263, GLY264, GLY319, GLY329, GLY342, GLY422, GLY444, GLY506
Mutated	W286A	1631	2	83.24	14.98	1.78	GLY14, GLY15, GLY26, GLY27, GLY43, GLY58, GLY121, GLY126, ALA127, GLY154, GLY169, GLY201, SER203, GLY230, GLY256, GLY261, GLY263, GLY264, GLY319, GLY329, GLY342, GLY422, GLY444, GLY523
Mutated	W286A	1631	3	82.5	16	1.5	GLY14, GLY15, GLY26, GLY27, GLY43, GLY58, TYR72, GLY126, ALA127, GLY154, GLY163, GLY201, GLY230, GLY260, GLY261, GLY263, GLY264, GLY319, GLY329, GLY342, GLY422, GLY444, GLU450, GLY506
Mutated	W86A	1631	1	82.63	15.79	1.58	GLY14, GLY15, GLY26, GLY27, GLY43, GLY58, GLY126, ALA127, GLY154, GLY201, SER203, GLY230, GLY256, GLY261, GLY264, GLY319, GLY329, GLY342, GLY422, GLY444, GLU450
Mutated	W86A	1631	2	82.43	15.87	1.7	GLY14, GLY15, GLY26, GLY27, GLY43, GLY58, GLY126, GLY154, GLY201, SER203, GLY230, GLY260, GLY261, GLY263, GLY264, GLY319, GLY329, GLY342, GLY422, GLY444, GLU450, GLY523
Mutated	W86A	1631	3	82.79	15.48	1.73	GLY14, GLY15, GLY26, GLY27, GLY43, GLY58, GLY79, GLY126, ALA127, GLY154, GLY169, SER203, GLY230, GLY260, GLY261, GLY263, GLY264, GLY319, GLY329, GLY342, GLY422
Wild	None	1631	1	83.28	14.82	1.89	GLY14, GLY15, GLY26, GLY27, GLY43, GLY58, GLY120, GLY122, GLY126, ALA127, GLY154, SER203, GLY230, GLY260, GLY261, GLY263, GLY264, GLY319, GLY329, GLY342, GLY422
Wild	None	1631	2	82.62	15.24	2.14	GLY14, GLY15, GLY26, GLY27, GLY43, GLY58, GLY120, GLY126, GLY154, GLY201, SER203, GLY230, GLY256, GLY260, GLY261, GLY263, GLY319, GLY329, GLY342, GLY422, GLY448, GLY523
Wild	None	1631	3	82.16	15.91	1.93	GLY14, GLY15, GLY26, GLY27, GLY43, GLY58, GLY120, GLY126, GLY154, GLY201, SER203, GLY230, GLY256, GLY261, GLY264, GLY319, GLY329, GLY342, GLY422, GLY444, GLY523
